# Psychological, social factors, and smoking behavior mediated the effects of cannabis use on personality disorders: A Mendelian randomization study

**DOI:** 10.3389/fpsyt.2025.1411587

**Published:** 2025-05-15

**Authors:** Yao Ni, Juanmei Li, Zitian Tang, Youqian Zhang, Yanyan Feng

**Affiliations:** ^1^ Department of Dermatovenereology, Chengdu Second People’s Hospital, Chengdu, Sichuan, China; ^2^ Department of Gynecology, Guang’anmen Hospital, China Academy of Chinese Medical Sciences, Beijing, China; ^3^ Health Science Center, Yangtze University, Jingzhou, Hubei, China

**Keywords:** cannabis use, Mendelian randomization, personality disorder, causality, mental disease

## Abstract

**Background:**

Rapid changes in attitudes, legality, and patterns of cannabis use (CU) underscore the importance of understanding its impact on mental health. Although links between CU and personality disorders (PDs) are documented, their causality remains uncertain.

**Methods:**

Employing Genome-Wide Association Studies (GWAS) data, this study investigated the causal relationship between cannabis use disorder (CUD) and lifetime cannabis use (LCU) with 9 types of PD risk through Mendelian randomization (MR) analysis. The primary method was the inverse variance weighted (IVW) method, supplemented by multivariable MR to assess direct effects independent of mental, social, and substance use factors, and mediation MR to explore mediating factors.

**Results:**

Corrections for the false discovery rate revealed significant causal associations between CUD and an increased risk of emotionally unstable PD (EUPD; OR_IVW_ = 1.228, 95% CI 1.069–1.411), overall PD (OR_IVW_ = 1.186, 95% CI 1.065–1.321), and schizoid PD (SPD; OR_IVW_ = 1.644, 95% CI 1.131–2.390). Mediation analysis identified schizophrenia (SCZ), major depressive disorder (MDD), neuroticism, and smoking initiation (SmkInit) as shared mediating factors between CUD and both EUPD and overall PD, with an additional mediating factor, household income (HI), specific to the CUD-to-overall PD pathway. In contrast, no mediating factors were found between CUD and SPD. Notably, a bidirectional causal relationship was observed between overall PD and CUD (OR_IVW_ = 1.399, 95% CI 1.033–1.895). Suggestive evidence indicated a causal link between lifetime cannabis use (LCU) and overall PD risk (OR_IVW_ = 1.074, 95% CI 1.008–1.146).

**Conclusion:**

This study offers new insights into the potential impact of CU on the development and progression of various PDs, laying the groundwork for targeted interventions to mitigate its effects on mental health.

## Introduction

Personality disorders (PD) are characterized as mental health conditions marked by enduring patterns of maladaptive behavior, cognition, and internal experience (1). Globally prevalent, the average prevalence rate of PD is 7.8%, with higher rates observed in developed countries (2). In general, personality traits represent patterns of thinking, perceiving, reacting, and relating that are relatively stable over time. PD exist when these traits become so pronounced, rigid, and maladaptive that they impair work and/or interpersonal functioning. PD in general are pervasive, enduring patterns of thinking, perceiving, reacting, and relating that cause significant distress or functional impairment, and usually start to become evident during late adolescence or early adulthood, it is shaped through interaction between genes and environment. Individuals with PD frequently encounter interpersonal difficulties, substance use disorder (SUD), self-harming behaviors, diminished quality of life, and often endure significant emotional distress (3), with prevalence rates among psychiatric patients ranging from 40% to 60% (1, 3, 4). The biopsychosocial model of mental disorder takes into account the biological, psychological, and social factors that contribute to the development of the diseases (disorder). It emphasizes the importance of considering the influence of social and behavioral factors on biological disease/condition. This burden not only impacts the individuals diagnosed but also their families and the broader healthcare system. Typically identified during adolescence or adulthood, though sometimes as early as childhood, the behavioral patterns associated with PD pose significant challenges (5, 6). However, prevention remains challenging due to the complex interplay of genetic, environmental, and psychological factors influencing its onset. Consequently, identifying and understanding the risk factors for PD is crucial for developing effective prevention strategies.

Cannabis, primarily composed of Δ-9 tetrahydrocannabinol (THC) and cannabidiol (CBD), is the most widely used illicit drug worldwide (7). In recent years, with rapid changes in attitudes, legal status, and patterns of cannabis use (CU), several countries have legalized recreational CU, which is notably prevalent among adolescents. Adolescence represents a critical period for the development of substance-related psychiatric disorders and other comorbid mental health conditions, such as PD (8). Research indicates that cannabis use disorder (CUD) is the most common SUD among adolescents receiving treatment for SUDs (9). Numerous observational studies have identified an association between CU or CUD and an increased incidence of PD and certain subtypes, where mental health factors, socio-economic factors, and other substance uses may act as mediating factors, creating a bridging effect (10–16). Studies have found that cannabis dependence is associated with higher rates of PD and deficits in social support. The potential interrelationship between interpersonal dysfunction and CUD and the correlation between PD and CUD deserve further study (15). A twin study in the Norwegian general population found that genetics may play a role in CU, CUD, and some PD traits, but not others (12). A study of the association between PD traits and problematic CU in adolescents showed that multiple regression analyses showed that PD traits largely explained the variance in problematic CU symptoms. After adjusting for anxiety and depression symptoms, schizotypic and borderline personality traits were positively associated with symptoms of problematic CU (17). Hasin et al. reported associations between CUD and all PD, with strongest associations reported between CUD and dependent or antisocial PD. Highest proportion of cannabis user were in those with a Cluster B PD (antisocial; borderline; histrionic; narcissistic) compared to Cluster A (paranoid; schizoid; schizotypal), or Cluster C (avoidant; dependent; antisocial) (18). However, there were no strong associations between CU and other later psychiatric disorders, suggesting that cannabis users with a PD may only be at significantly elevated risk for additional substance use disorder. Individuals with any PD are significantly more likely to have a past-year CUD than those without. Borderline PD has the strongest association, compared to antisocial and schizotypal, and these findings are consistent with a twin study, as well as numerous other studies. Genetic and environmental correlations between PD traits and CU suggest that genetic risks in borderline and antisocial PD traits accounted for significant variance in CU, however not for schizoid or dependent PD traits (19). There is no doubt that there is an association between PD and substance use/addiction (CU/addiction). People with PD are at increased risk of other mental disorders, including substance use and addiction. In addition to this, CU in children and adolescents can be a “symptom” of other disorders. However, due to inherent limitations in traditional research, the causal relationship has not been fully established, and there is a lack of systematic research causally linking CU with PD and all its subtypes.

Although randomized controlled trials (RCTs) are considered the gold standard for establishing causal relationships, large-scale RCTs are often hindered by high costs, time and resource intensity, and sometimes impracticality or ethical concerns. Mendelian randomization (MR), a genetic analysis technique akin to RCTs, has emerged as an alternative research method capable of overcoming these limitations. It utilizes genetic variations identified in Genome-Wide Association Studies (GWAS) as instrumental variables (IVs) to determine the causal impact of risk factors (or exposures) on outcomes (20). Given that alleles related to exposures are randomly assigned at birth (21), MR can circumvent issues of confounding and reverse causation, which frequently challenge traditional epidemiological studies (22). This study aims to employ MR analysis to explore the bidirectional causal relationship between CU and PD, offering insights that could inform prevention and intervention strategies.

## Materials and methods

### Study design

**Figure 1:**
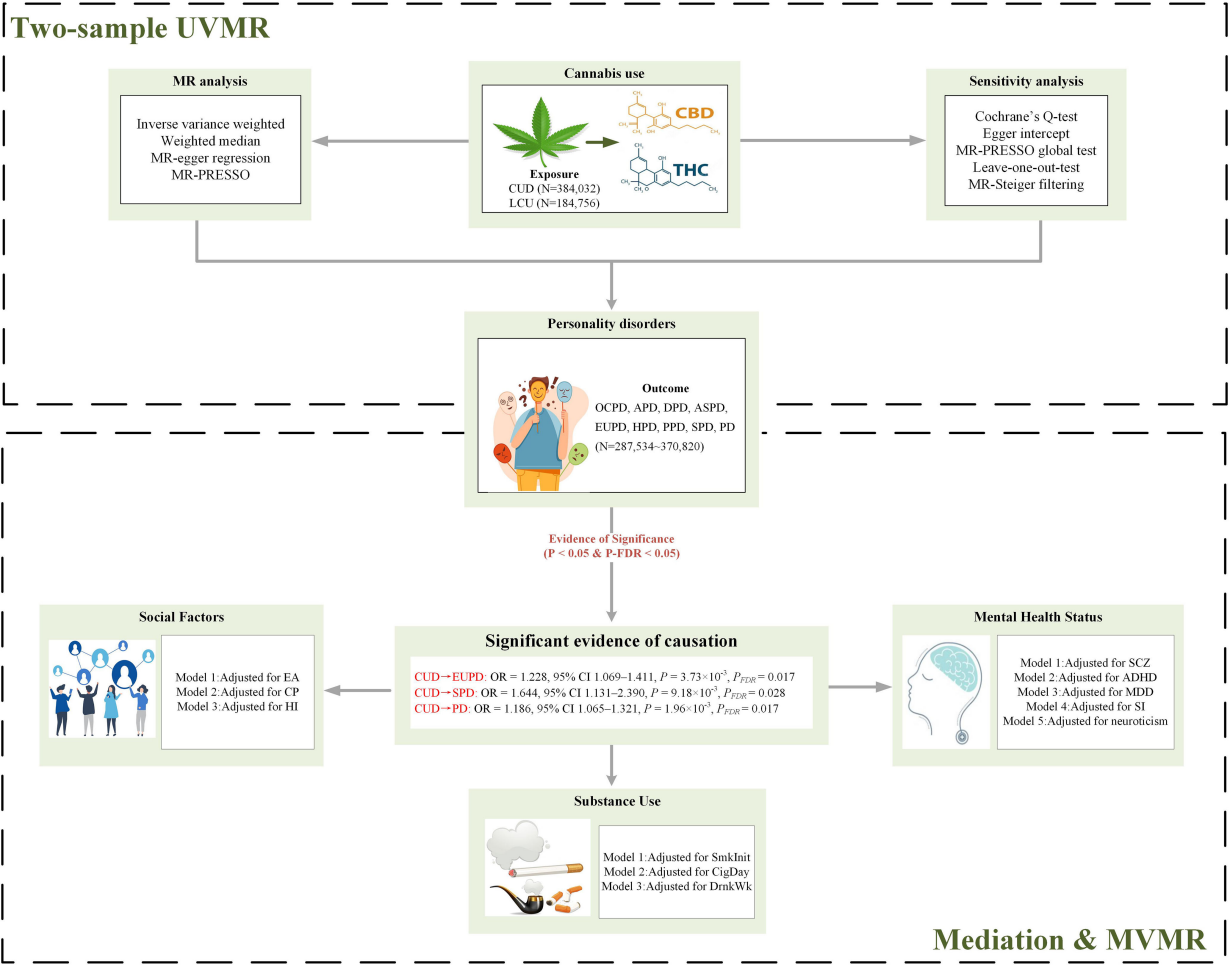
Flowchart of Mendelian randomization analysis. PD, personality disorders; OCPD, anankastic (obsessive–compulsive) personality disorder; APD, anxious personality disorder; DPD, dependent personality disorder; ASPD, antisocial personality disorder; EUPD, emotionally unstable personality disorder; HPD, histrionic personality disorder; PPD, paranoid personality disorder; SPD, schizoid personality disorder; CUD, cannabis use disorder; LCU, lifetime cannabis use; SCZ, schizophrenia; ADHD, attention deficit hyperactivity disorder; CigDay, cigarettes per day; SmkInit, smoking initiation; DrnkWk, alcoholic drinks per week; EA, education attainment; CP, cognitive performance; HI, household income; SI, social Isolation; MDD, major depressive disorder; MR, Mendelian randomization; MVMR, multivariable MR; UVMR, univariable MR; THC, Δ-9 tetrahydrocannabinol; CBD, cannabidiol; FDR, false discovery rate; MR-PRESSO, MR Pleiotropy Residual Sum and Outlier.

This study utilizes summary-level data from publicly available GWAS to conduct MR analysis. To investigate the causal relationship between CU and PD, an initial bidirectional univariable MR (UVMR) analysis was performed, followed by a multivariable MR (MVMR) to assess the independence of the causal association from three major confounding factors: mental, social, and substance use. Further mediation MR analysis explored whether confounding phenotypes exhibit a bridging effect. The exposure IVs employed for analysis adhere to the three core assumptions of MR: i) the genetic instruments designated as IVs must be strongly associated with the exposure; ii) these genetic instruments should not be confounded with any potential confounding factors; iii) the influence of the genetic instruments on the outcome must be mediated solely through their interaction with the exposure, not through other outcome phenotypes (23). A flowchart of the MR analysis process is presented in **Figure 1**.

### Data source

As a secondary analysis of publicly available data, this study did not require further ethical review. Ethical approval, participant informed consent, and eligibility criteria for each original GWAS can be found in their respective publications. CU encompasses CUD and lifetime cannabis use (LCU), derived from the Psychiatric Genomics Consortium (PGC) (24) and the International Cannabis Consortium (ICC) (25), respectively. Nine PD phenotypes were obtained from the FinnGen consortium’s R9 version data (26). Confounding and mediating phenotypes for further analysis were categorized into three major groups: mental health, social factors, and substance use. Schizophrenia (SCZ), attention deficit hyperactivity disorder (ADHD), and major depressive disorder (MDD) were sourced from PGC (27–29); social isolation (SI), neuroticism, and household income (HI) from the UK biobank (UKB) (30); educational attainment (EA) and cognitive performance (CP) from the Social Science Genetic Association Consortium (SSGAC) (31); smoking initiation (SmkInit), cigarettes per day (CigDay), and drinks per week (DrnkWk) from the GWAS and Sequencing Consortium of Alcohol and Nicotine use (GSCAN) (32). All sample populations originated from Europe, with no sample overlap as determined through computation. **Table 1** presents all datasets used for analysis, including diagnostic criteria, sample sizes, and sources.

**Table 1:** Detailed information of data sources.

Phenotype	Definition/diagnostics	Ref	Consortium	Ancestry	Participants
OCPD	ICD-10-F60.6,ICD-8-3014	36653562	FinnGen	EUR	1,032 cases/366,637 controls
APD	ICD-10-F60.6,ICD-9-3018C	36653562	FinnGen	EUR	534 cases/366,637 controls
DPD	ICD-10-F60.7,ICD-9-3016,ICD-8- 3016	36653562	FinnGen	EUR	610 cases/366,637 controls
ASPD	ICD-10-F60.2,ICD-9-3017,ICD-8-3017	36653562	FinnGen	EUR	467 cases/366,637 controls
EUPD	ICD-10-F60.3,ICD-9-3018D,ICD-8-3013	36653562	FinnGen	EUR	4,183 cases/366,637 controls
HPD	ICD-10-F60.4,ICD-9- 3015,ICD-8- 3015	36653562	FinnGen	EUR	142 cases/366,637 controls
PPD	ICD-10-F60.0,ICD-9-3010,ICD-8-3010	36653562	FinnGen	EUR	559 cases/366,637 controls
SPD	ICD-10-F60.1,ICD-9-3012,ICD-8-3012	36653562	FinnGen	EUR	550 cases/366,637 controls
PD	ICD-10-F60,F61,ICD-9-31[0|2|4]|315A|31[6-8],ICD-8-30100|301[2-9]	36653562	FinnGen	EUR	10,012 cases/277,522 controls
CUD	DSM-III-R, DSM-IV, DSM-5 or ICD10	33096046	PGC	EUR	17,068 cases and 357,219 controls
LCU	Selfreporting ever using cannabis in a life-time	30150663	ICC	EUR	184,756 individuals
Adjustment of the model					
SCZ	DSM, ICD-10,diagnostic interviews, medical records or hospital registers	35396580	PGC	EUR	52,017 cases and 75,889 controls
ADHD	DSM-III-R, DSM-5, DSM-IV, ICD-10 or self-reported	30478444	PGC	EUR	20,183 cases and 35,191 controls
MDD	Self-report, hospital records or structured interviews	30718901	PGC	EUR	170,756 cases/329,443 controls
SI	Questionnaire: Yes/No feel lonely	36402876	UKB	EUR	58,752 cases/273,511 controls
Neuroticism	Questionnaire: 12 neurotic behaviors	36402876	UKB	EUR	393411 individuals
EA	Years of education	30038396	SSGAC	EUR	1,131,881 individuals
CP	Respondent’s score on a test of verbal cognition	30038396	SSGAC	EUR	257,841 individuals
HI	Questionnaire: household income	36402876	UKB	EUR	397,751 individuals
SmkInit	Ever/never	30643251	GSCAN	EUR	311,629 cases and 321,173 controls
CigDay	Heaviness of smoking	30643251	GSCAN	EUR	337,334 individuals
DrnkWk	Alcohol consumption	30643251	GSCAN	EUR	335,394 individuals

ICC, International Cannabis Consortium; PD, personality disorders; OCPD, anankastic (obsessive–compulsive) personality disorder; APD, anxious personality disorder; DPD, dependent personality disorder; ASPD, antisocial personality disorder; EUPD, emotionally unstable personality disorder; HPD, histrionic personality disorder; PPD, paranoid personality disorder; SPD, schizoid personality disorder; CUD, cannabis use disorder; LCU, lifetime cannabis use; EUR, European; SCZ, schizophrenia; PGC, Psychiatric Genomics Consortium; ADHD, attention deficit hyperactivity disorder; GSCAN, GWAS and Sequencing Consortium of Alcohol and Nicotine use; CigDay, cigarettes per day; SmkInit, smoking initiation; DrnkWk, alcoholic drinks per week; EA, education attainment; SSGAC, Social Science Genetic Association Consortium; CP, cognitive performance; HI, household income; SI, social Isolation; MDD, major depressive disorder.

### Selection of genetic instrumental variables

This MR study implemented a rigorous screening process for single nucleotide polymorphisms (SNPs) with the following criteria: (1) SNPs were initially screened at the genome-wide significance level (*P* < 5×10^-8^) for association with the exposure. However, due to the absence of genome-wide significant SNPs for CUD, a more relaxed threshold (*P* < 5×10^-7^) was adopted based on previous MR studies on CUD (33). In reverse MR analysis, an even more relaxed threshold (*P* < 1×10^-5^) was used to ensure a sufficient number of IVs, thereby enhancing statistical power. (2) A stringent linkage disequilibrium (LD) clumping method (r^2^ < 0.001 within 10,000 kb window base on 1000 Genomes Phase 3) was employed to identify independent SNP loci, minimizing bias due to LD. (3) MR-Steiger filtering was utilized to exclude SNPs with potential reverse causation (34). (4) The F-statistic *[F=((n −k−1)/k)(R^2^/(1− R^2^))]*, where R^2^ is the variance in exposure accounted for by the SNPs, *k*=1 reflects a single SNP analysis, and N represents the GWAS sample size) for all SNPs was required to be greater than 10 to avoid bias from weak IVs (35). (5) Proxy SNPs (r^2^ > 0.8) were not used to ensure the precision of the results.

### Statistical analyses

#### Primary MR analysis

For the UVMR study, the Wald ratio test was employed initially for individual IVs. Following this, the multiplicative random-effects inverse-variance-weighted (IVW) method was implemented for the causative assessment of multiple IVs (comprising 2 or more). This approach was further enhanced by the incorporation of both MR-Egger and weighted median techniques. The weightage in IVW bears a direct relation to each SNP’s Wald ratio estimate and an inverse correlation to the variance estimate of each SNP’s Wald ratio (36). When all genetic markers are adjudged valid, IVW provides consistent and efficient estimates. Conversely, the weighted median method stands out when over half of the genetic markers are deemed questionable, and the MR-Egger approach is opted for when the entirety of genetic markers is deemed untenable (37). A stringent adjustment for multiple comparisons was implemented via the false discovery rate (FDR). Following this adjustment, a *P*-value falling below 0.05 was considered indicative of a significant causal relationship. However, instances where the raw *P*-value was below 0.05, but the FDR-adjusted *P*-value exceeded this threshold, were merely regarded as tentative.

Given the potential confounding impact of factors on the path from exposure to outcome, subsequent MVMR analyses were orchestrated. The aim was to accurately quantify the direct causative repercussion of exposure on the result. When juxtaposed with the UVMR paradigm, the primary supposition of MVMR revolves around genetic variability associating with one or more exposures, while the succeeding assumptions harmonize with the UVMR framework (38). To further explore the potentially modifiable mediating factors between cannabis use and PD, we conducted a two-step mediation MR analysis. This approach extends traditional MR by incorporating mediation pathways, leveraging genetic variants as instrumental variables to minimize confounding and reverse causality often encountered in observational mediation studies. In this framework, three core causal effects were considered: (1) the direct effect of cannabis use on PD, independent of the mediator; (2) the indirect effect transmitted through the mediator; and (3) the total effect, representing the combined influence of both direct and indirect pathways. The proportion of mediation was quantified by the ratio of the indirect effect to the total effect, which reflects the extent to which the mediator accounts for the observed association. This strategy enhances causal inference and elucidates biological pathways, offering valuable insights for the development of precise and targeted interventions.

#### Power calculation

Given that most genetic variants predict only a small fraction of phenotypic variance, statistical power is considered one of the primary challenges in MR study (39). This MR study further calculates R² (the proportion of explained variance) and statistical power. R² was calculated employing the formula 2×MAF×(1-MAF)×beta², wherein MAF represents the minor allele frequency of each specified SNP (40). These cumulative values yielded the coefficient crucial for the estimation of statistical power. The assessment of statistical power was conducted using the mRnd platform, available at (https://shiny.cnsgenomics.com/mRnd/) (39).

#### Sensitivity analysis

Within the framework of the UVMR analysis, several tests were conducted to validate rigor and authenticity. The heterogeneity of the selected genetic variants was assessed using Cochran’s Q test, wherein a *P*-value of < 0.05 indicated pronounced discrepancies among the scrutinized SNPs (41). Employing the MR-Egger regression (42), the investigation discerned the potential for directional pleiotropy within the MR context. The MR-Egger’s intercept, bearing a *P*-value of < 0.05, signified consequential directional pleiotropy (43). However, causal estimates obtained through the MR-Egger method are subject to inherent limitations that could introduce bias and potentially inflate the type I error rate, thereby requiring careful interpretation.The MR Pleiotropy Residual Sum and Outlier (MR-PRESSO) approach was deployed to identify probable outliers and to delve into horizontal pleiotropy, which was inferred when the global P-value was less than 0.05 (44). By excluding such outliers, the refinement of our data correction was realized. An ensuing leave-one-out analysis shed light on the impact exerted by singular SNPs on the collective outcomes (45).

#### Data analysis software and packages

All statistical analyses were performed using R software (version 4.2.2), TwoSampleMR (version 0.5.6), MR-PRESSO (version 1.0), MVMR (version 0.4), MendelianRandomization (version 0.9.0) were analyzed.

## Results

### Genetic instrument selection and calculation of power statistics

Regarding the CU phenotype, this study initially acquired 12 SNPs related to CUD from the PGC and 8 SNPs related to LCU from the ICC. Through filtering, the number of IVs utilized in both forward and reverse UVMR analyses ranged from 2 to 47, explaining genetic variance from 0.59% to 36.18% (**Supplementary Table S1**). All IVs were filtered through the Steiger test, fulfilling the III assumption of MR analysis. Additionally, MR-PRESSO analysis was strictly employed to eliminate outliers, reducing bias introduced by horizontal pleiotropy. The F-statistics calculated for each IV exceeded 10, with averages ranging from 239 to 37,077, significantly minimizing bias due to weak instrumental variables. Moreover, the study possessed sufficient statistical power to detect positive causal associations. Specifically, in forward UVMR analyses, with ORs of 1.228, 1.644, and 1.186, we had statistical powers of 92%, 94%, and 95%, respectively, to detect associations of CUD with emotionally unstable PD (EUPD), schizoid PD (SPD), and PD. With an OR of 1.399, there was an 85% statistical power to detect an association of LCU with PD. In reverse UVMR analyses, an OR of 1.074 provided a 95% statistical power to detect an association of PD with CUD (**Supplementary Table S1**). This further strengthens the robustness of the causal associations.

### Causal association of CU with PD and their subtypes

**Figure 2:**
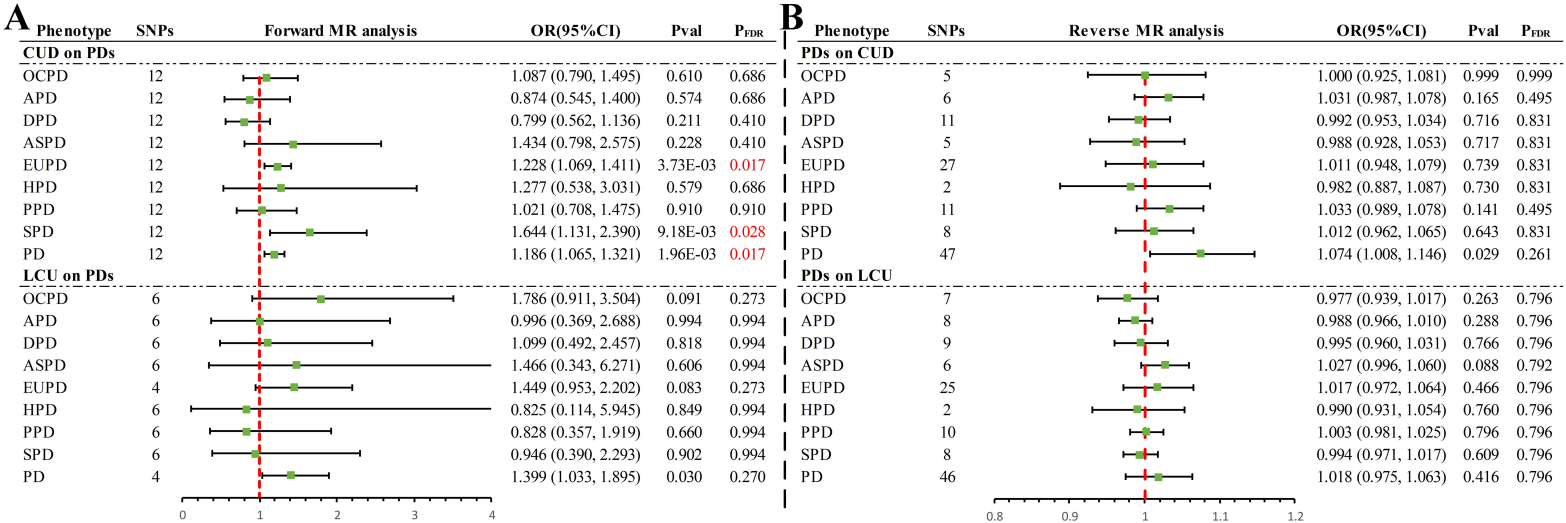
Summary of results of IVW methods for forward and reverse univariate Mendelian randomization analysis. **(A)** Forward MR analysis **(B)** Reverse MR analysis. PD, personality disorders; OCPD, anankastic (obsessive–compulsive) personality disorder; APD, anxious personality disorder; DPD, dependent personality disorder; ASPD, antisocial personality disorder; EUPD, emotionally unstable personality disorder; HPD, histrionic personality disorder; PPD, paranoid personality disorder; SPD, schizoid personality disorder; CUD, cannabis use disorder; LCU, lifetime cannabis use; SNP, single nucleotide polymorphisms; MR, Mendelian randomization; FDR, false discovery rate.

In the forward UVMR analysis (**Figure 2**), after correction for FDR, the IVW analysis revealed three significant and one suggestive causal association. Specifically, a genetic predisposition to CUD was significantly associated with an 18.6% increase in the incidence of PD (OR = 1.186, 95% CI 1.065–1.321, *P* = 1.96×10^-3^, *P_FDR_* = 0.017) per standard deviation (SD) increase. LCU also provided suggestive evidence of an increase in PD incidence (OR = 1.399, 95% CI 1.033–1.895, *P* = 0.030, *P_FDR_* = 0.270). Additionally, significant causal associations were found between CUD and an increase in the incidence rates of EUPD (OR = 1.228, 95% CI 1.069–1.411, *P* = 3.73×10^-3^, *P_FDR_* = 0.017) and SPD (OR = 1.644, 95% CI 1.131–2.390, *P* = 9.18×10^-3^, *P_FDR_* = 0.028) by 22.8% and 64.4%, respectively, with the weighted median method providing consistent results (EUPD: OR = 1.327, 95% CI 1.083–1.627, *P* = 0.006; SPD: OR = 1.767, 95% CI 1.098–2.845, *P* = 0.019). However, no causal associations were found between CUD or LCU and other PD subtypes (*P* > 0.05 & *P_FDR_* > 0.05) (**Supplementary Table S2**).

In the reverse UVMR analysis (**Figure 2**), a bidirectional causal association was identified between CUD and PD. Specifically, a genetically predicted increase of one SD in PD was suggestively associated with a 7.4% increase in the incidence of CUD (OR = 1.074, 95% CI 1.008–1.146, *P* = 0.029, *P_FDR_* = 0.261). No causal associations were found between other PD phenotypes and CUD or LCU (*P* > 0.05 & *P_FDR_* > 0.05) (**Supplementary Table S2**).

Sensitivity analyses revealed no evidence of horizontal pleiotropy via MR-Egger (*P* > 0.05) (**Supplementary Table S3**). With few exceptions (**Supplementary Table S3**), Cochran’s Q statistic detected no heterogeneity (*P* > 0.05), and MR-PRESSO found no evidence of horizontal pleiotropy (*P* > 0.05), excluding two outliers (rs10085617, rs9773390) in the analysis of LCU’s effect on EUPD and PD. Leave-one-out analysis indicated that the causal association in the analysis of LCU’s effect on PD was driven by a single SNP (rs2875907) (**Supplementary Figure S1**), suggesting that the results should be considered suggestive evidence. The remaining causal associations remained robust, unaffected by any single SNP (**Supplementary Figure S1–S4**). Funnel plots-maintained symmetry (**Supplementary Figure S5–S8**). Scatter plots clearly illustrated the direction of each analysis (**Supplementary Figure S9–S12**), while forest plots provided the contribution of each IV (**Supplementary Figure S13–S16**).

### Adjusting for Confounding Factors and Conducting Mediation MR Analysis

**Figure 3:**
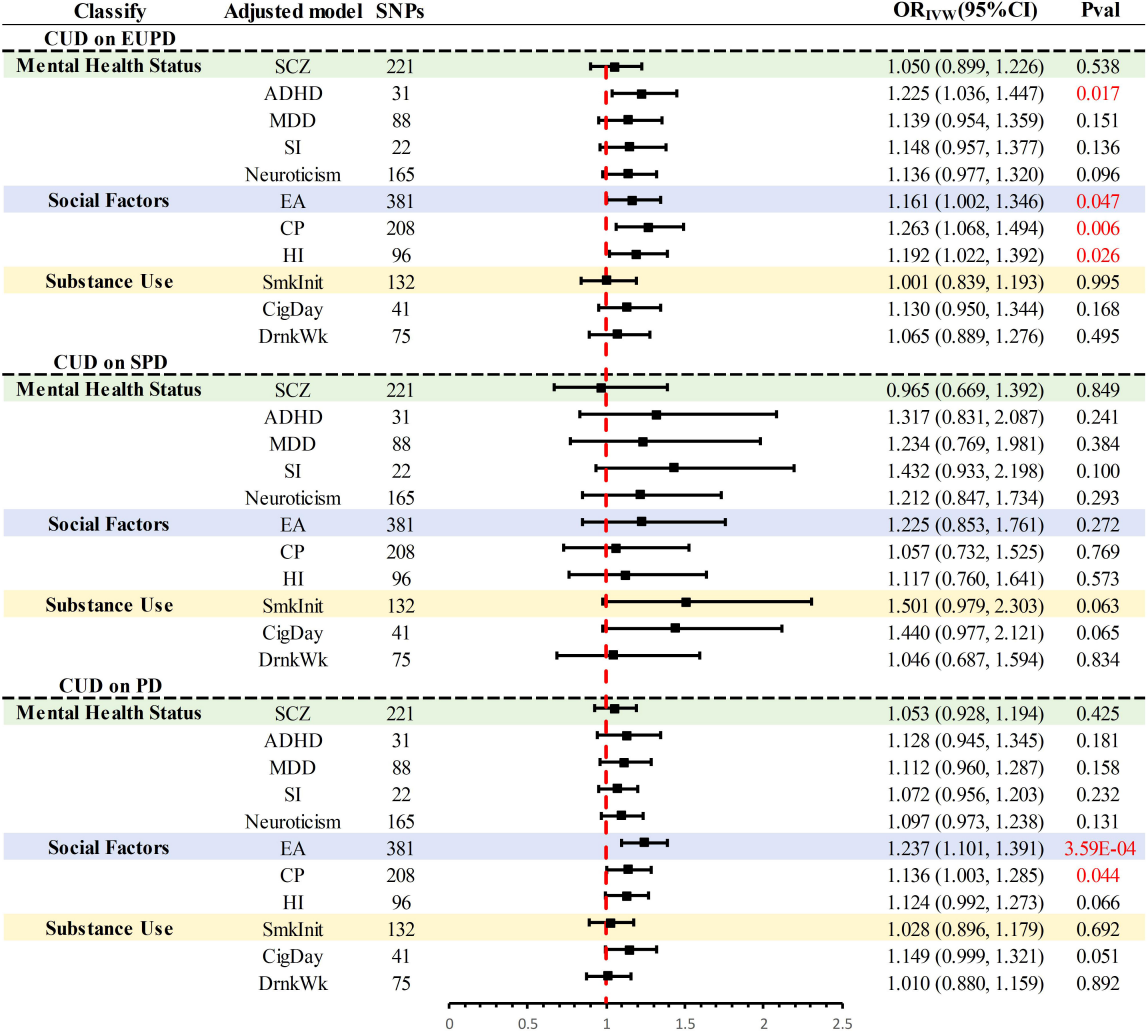
Summary of multivariate MR results for significant causal association. PD, personality disorders; EUPD, emotionally unstable personality disorder; SPD, schizoid personality disorder; CUD, cannabis use disorder; SCZ, schizophrenia; ADHD, attention deficit hyperactivity disorder; CigDay, cigarettes per day; SmkInit, smoking initiation; DrnkWk, alcoholic drinks per week; EA, education attainment; CP, cognitive performance; HI, household income; SI, social Isolation; MDD, major depressive disorder; MR, Mendelian randomization.

In the UVMR analysis, significant causal associations warranted further investigation through MVMR to assess whether the causal effects of CUD on PD and its subtypes were independent of three major confounding factors (**Figure 3**). Within the realm of mental health factors, evidence was found for a direct impact of CUD genetic predisposition on the incidence of EUPD (*P* = 0.017), independent of ADHD. Regarding social factors, evidence was discovered for a direct influence of CUD genetic susceptibility on the incidence rates of EUPD and PD, independent of EA and CP (*P* < 0.05). Additionally, direct evidence of CUD’s impact on EUPD, independent of HI (*P* = 0.026), was identified. After adjusting in the remaining models, the causal relationship between CUD and PD and its subtypes was no longer significant (*P* > 0.05).

Further mediation MR analysis (**Supplementary Table S4**, **Supplementary Figure S17**) indicated common mediating factors in the analysis of CUD’s effects on EUPD and PD, including SCZ, MDD, neuroticism, and SmkInit, with neuroticism explaining the largest mediation effect (EUPD: 21.50%; PD:27.95%) (**Table 2**). Additionally, a mediating effect of HI (Mediation effect: 15.33%) was found in the analysis of CUD’s impact on PD (**Table 2**).

**Table 2:** All positive results of the mediation effect analysis of CU on PD.

Phenotype	Mediator	Total effect Effect size (95% CI)	Direct effect Effect size (95% CI)	Mediation effect		
				Effect size (95% CI)	IE div TE(%)	*P*
CUD on EUPD	SCZ	0.205 (0.067, 0.344)	0.185 (0.045, 0.325)	0.205 (0.067, 0.344)	9.81%	0.030
	MDD	0.205 (0.067, 0.344)	0.163 (0.020, 0.307)	0.042 (0.004, 0.080)	20.43%	0.028
	Neuroticism	0.205 (0.067, 0.344)	0.161 (0.020, 0.303)	0.044 (0.016, 0.072)	21.50%	0.002
	SmkInit	0.205 (0.067, 0.344)	0.174 (0.033, 0.316)	0.031 (0.005, 0.057)	15.12%	0.019
CUD on PD	SCZ	0.171 (0.063, 0.278)	0.142 (0.031, 0.252)	0.029 (0.005, 0.053)	16.88%	0.019
	MDD	0.171 (0.063, 0.278)	0.126 (0.011, 0.240)	0.045 (0.007, 0.083)	26.32%	0.020
	Neuroticism	0.171 (0.063, 0.278)	0.123 (0.011, 0.234)	0.048 (0.020, 0.075)	27.95%	0.001
	HI	0.171 (0.063, 0.278)	0.144 (0.034, 0.254)	0.026 (0.005, 0.048)	15.33%	0.015
SmkInit	0.171 (0.063, 0.278)	0.153 (0.044, 0.262)	0.017 (0.001, 0.034)	10.20%	0.033

CU, cannabis use; PD, personality disorders; EUPD, emotionally unstable personality disorder; CUD, cannabis use disorder; PD, personality disorder; SCZ, schizophrenia; SmkInit, Smoking initiation; HI, household income; MDD, major depressive disorder; IE div TE, Indirect Effect divided by Total Effect; CI, confidence interval.

## Discussion

This study conducted a comprehensive MR analysis to explore the genetic predisposition of CU and its causal relationship with various PD risks. The model genetically predicted that CUD would increase the risk of EUPD, SPD, and overall PD. On the contrary, overall PD can increase the risk of CUD, and there was a two-way causal relationship between CUD and overall PD. In addition, genetic evidence suggested that LCU patients get a higher risk of overall PD, while no clear causal relationship has been found between overall PD and LCU. Further analysis pinpointed five mediating factors. The implications of these findings will be discussed from the perspectives of neurobiological mechanisms and social factors.

The primary active component of cannabis, THC, affects key neurotransmitter systems in the brain by acting on cannabinoid receptors, especially CB1 receptors. This action influences emotion regulation, stress response, reward perception, and social behavior, as indicated by extensive research (46, 47). Long-term or heavy CU can lead to dysfunction of these systems, cerebellar hypoactivation, and structural brain changes, particularly in the white matter, prefrontal cortex, hippocampus, and amygdala (48–51). Such changes may trigger issues with emotional stability, social skills, and impulse control, increasing the risk of developing disorders like EUPD and SPD. Further mediation MR analysis revealed several mental health mediating factors. SCZ and MDD reflect a broader spectrum of psychological vulnerability, exhibiting a complex bidirectional relationship with CU. Numerous MR studies have established a causal link between CU and an increased incidence of these psychiatric conditions (52–54), while our reverse MR analysis further reveals a bidirectional causal relationship between CUD and PD. This reciprocal relationship suggests that individuals with these conditions may be more inclined to use cannabis as a form of self-medication. Inappropriate treatment may lead to cannabis dependence, exacerbating mental health issues and creating a vicious cycle. A systematic review by Jerome Sarris’ team provides consistent evidence and emphasizes the need for clinicians to consider a range of prescription and occupational safety factors (55). Additionally, research by Kaeli Zimmermann’s team indicates that individuals with high neuroticism are more likely to use cannabis to alleviate negative emotions (56). Frequent cannabis users show impaired emotional reevaluation and increased neural activity in areas associated with emotion regulation, indirectly increasing the risk of PDs. Therefore, interventions targeting these mediating factors can effectively reduce PD risk from a genetic perspective, promoting mental health and social well-being.

Dongze Chen’s MR analysis has established a bidirectional causal association between CU and education, highlighting the potential adverse effects of cannabis on individual educational and occupational achievements (33). This association suggests an increase in conflicts with family and society, leading to psychological stress and social isolation, particularly during adolescence (57). Notably, the level of HI reflects the socio-economic status of individuals and families, where lower income may limit resources for stress management and increase the psychological burden of living in disadvantaged socio-economic conditions, thereby elevating the risk of developing CUD and PD. Correction of SI and HI factors in MVMR analysis resulted in a non-significant causal relationship between CUD and PD, further validating these factors as critical in the development of PD. Smoking behavior may not only exist as a habit but also reflect deeper psychological and social dynamics. Adolescence, a crucial period for personality formation, social-psychological development, and self-identity exploration, may predispose individuals to experiment with substances such as cannabis and initiate smoking as a means to cope with social pressures, emotional disturbances, or identity exploration. This perspective aligns with Michael Windle’s emphasis on how adolescent delinquency, academic performance, and stressful life events can predict these trajectories, potentially increasing the risk of PD (58). Our mediation analysis underscores the mediating effect of smoking initiation, providing consistent evidence. Thus, smoking behavior and household income levels may be bridges connecting CUD and PD, both by increasing the likelihood of CU and through shared underlying mental health issues.

LCU, defined as having used cannabis at least once without implying continuous or frequent use, contrasts with CUD, which represents a severe dependency indicating regular, intensive, and prolonged consumption of cannabis. This distinction explains the differential outcomes of LCU and CUD on PD, where EUPD includes impulsive (F60.30) and borderline (F60.31) PDs. Hence, this MR study is consistent with previous epidemiological research, marking the first identification of a causal association between genetically predicted CUD and increased risk of SPD, highlighting the dose-response relationship previously noted in research on CU and mental illness (59). However, the absence of a positive causal association with other PD subtypes, beyond the nature of LCU versus CUD, may be attributed to three potential reasons: limitations in sample size for different PD subtype causality studies, individual responses to cannabis and PD development influenced by genetic factors, and the association of different PDs with distinct neurotransmitter systems and neural circuits in the brain. Further research and analysis are needed in the future.

Individual differences in the liability to CU and CUD appear to be linked to genetic risks correlated with antisocial and borderline PD traits. A cross-sectional survey of 1,419 twins at the Norwegian Institute of Public Health examined which combination of PDs trait scores best predicted CU and CUD, and estimated the magnitude and significance of genetic and environmental risks in PD traits shared with CU and CUD. The results found that genetic risk for antisocial and borderline PD traits explained 32-60% of the total variance of CU and CUD. Among them, the genetic risk of antisocial PD traits explained CU (56%) and CUD (43%). Borderline PD traits explained CU (32%) and CUD (60%). It can be seen that individual differences in cannabis use susceptibility and cannabis use disorder appear to be associated with genetic risk associated with antisocial and borderline personality disorder traits (12). These phenotypes and genotypes were found to have a strong impact on lifetime alcohol use and alcohol use disorders, suggesting that alcohol and CU and abuse share many of the same genetic and environmental risk factors (12). In results reported by Norwegian twins, we found that borderline and antisocial PD trait scores were also the strongest associations over time and across time for phenotypic and genotypic susceptibility to lifetime drinking and alcohol use disorders (60). This suggests that lifetime use and abuse of alcohol and cannabis are indexed by many of the same genetic and environmental risk factors. It has been hypothesized that early (adolescent) alcohol abuse and co-use of cannabis are causal risk factors for adult antisocial/psychotic personality by altering brain structure and function, as both usually occur simultaneously (61). Our findings appear to provide partial support for this hypothesis, suggesting a two-way relationship between alcohol/cannabis use and antisocial PD.

This study presents several strengths, notably being the first comprehensive MR analysis to establish the causal relationship between CU and PD. This is the first study to investigate all 9 PDs and explore associations with CU and use disorder within the context of genetics. It employs a range of sensitivity analyses to ensure robust results and utilizes MVMR and mediation MR analyses to explore multiple mediating factors, offering a broader perspective. However, the study also has limitations, including a population predominantly from Europe, reducing the diversity of the sample. Additionally, reliance on summary-level GWAS data limits subgroup analysis and further exploration of dose-response relationships.

## Conclusion

This MR study offers new insights into the potential impact of CU on the development and progression of various PDs, laying the groundwork for targeted interventions to mitigate its effects on mental health.

## Data Availability

The original contributions presented in the study are included in the article/Supplementary Material. Further inquiries can be directed to the corresponding author/s.
