# Supplementary material for: Psychological, social factors, and smoking behavior mediated the effects of cannabis use on personality disorders: A Mendelian randomization study
Source: Front Psychiatry. 2025 May 15;16:1411587. doi: 10.3389/fpsyt.2025.1411587 (PMC12119549; doi:10.3389/fpsyt.2025.1411587)
Supplement: Supplementary file 2 [file DataSheet2.docx]

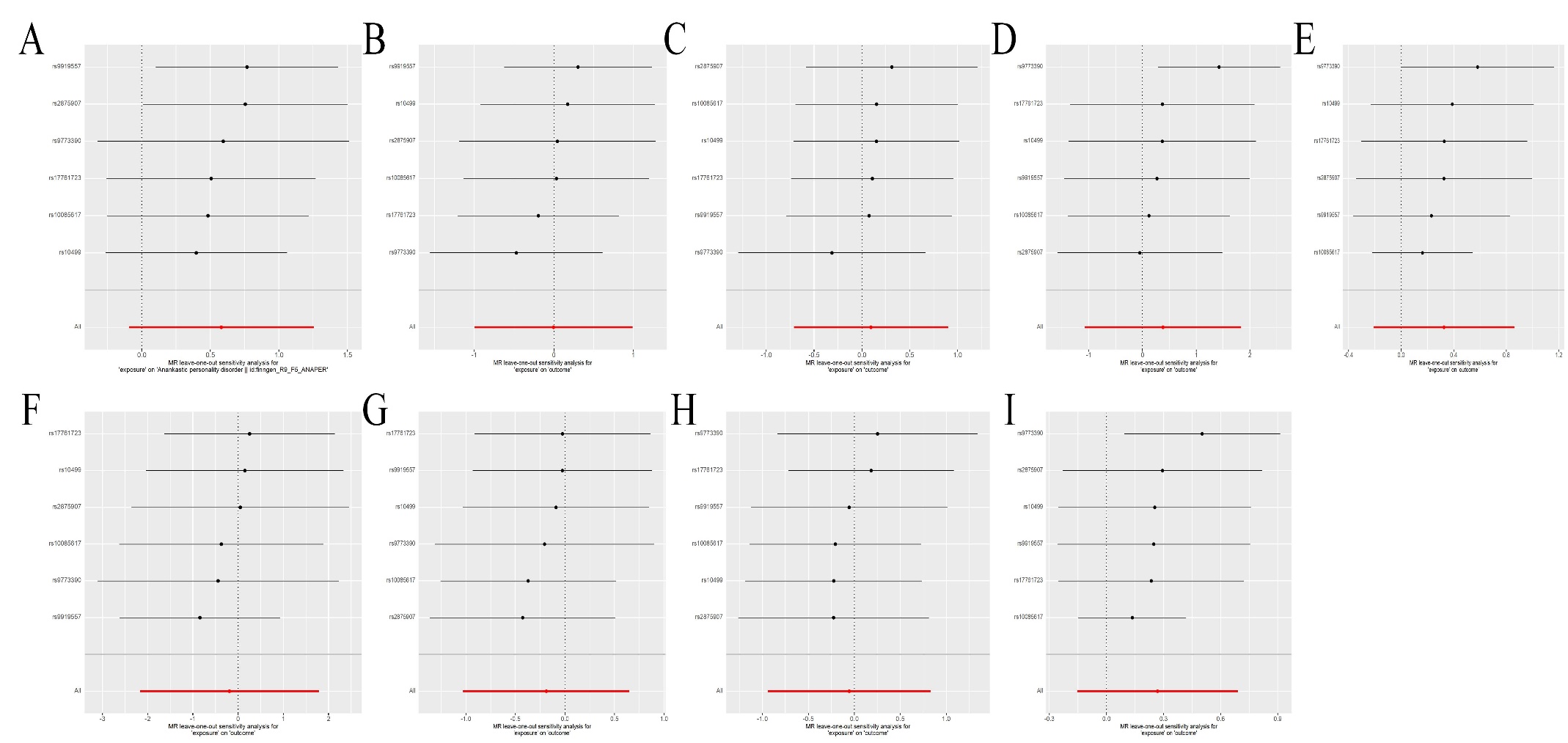


**Supplementary Fig. 1** Leave-one-out plot for MR analysis of LCU on PDs. (A) LCU on OCPD (B) LCU on APD (C) LCU on DPD (D) LCU on ASPD (E) LCU on EUPD (F) LCU on HPD (G) LCU on PPD (H) LCU on SPD (I) LCU on PD. PD, personality disorders; OCPD, anankastic (obsessive–compulsive) personality disorder; APD, anxious personality disorder; DPD, dependent personality disorder; ASPD, antisocial personality disorder; EUPD, emotionally unstable personality disorder; HPD, histrionic personality disorder; PPD, paranoid personality disorder; SPD, schizoid personality disorder; LCU, lifetime cannabis use.


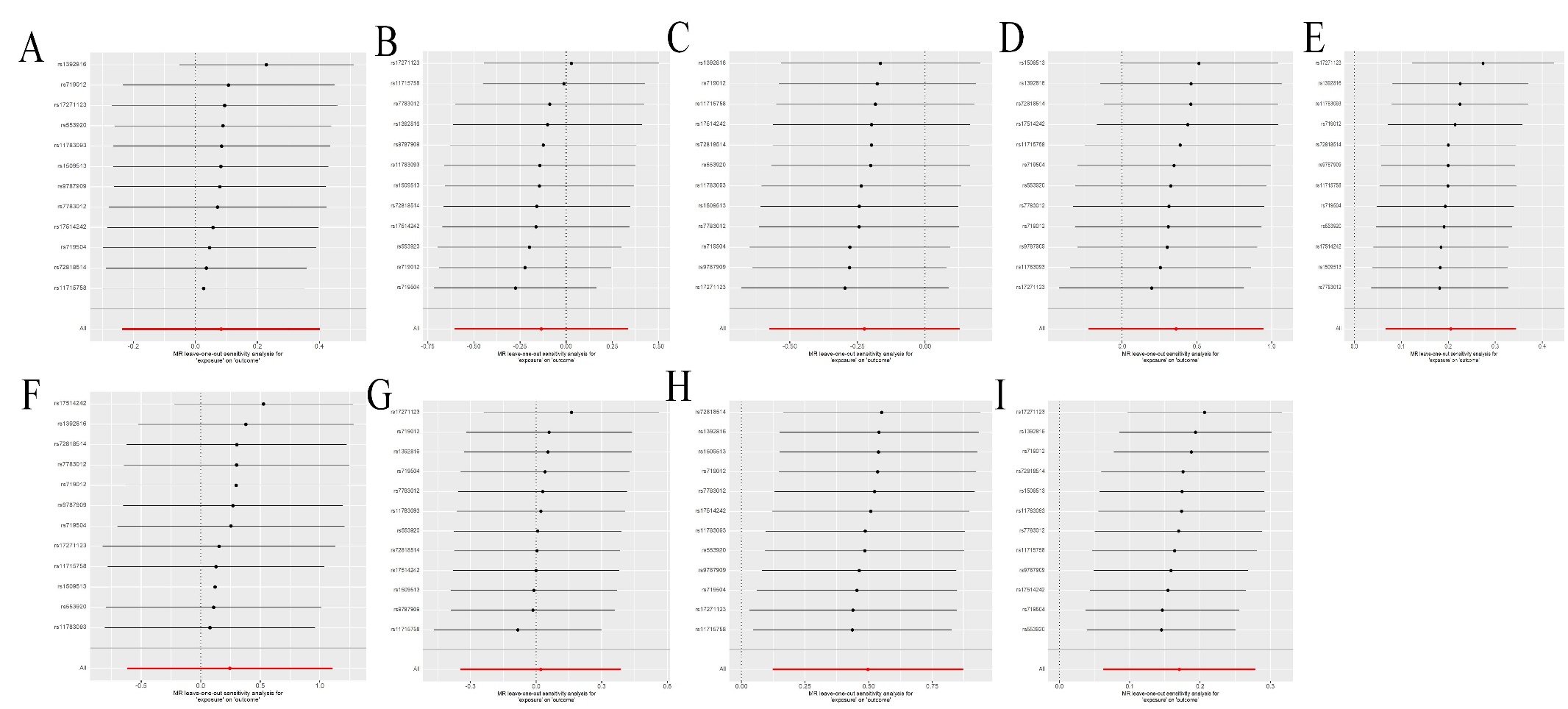


**Supplementary Fig. 2** Leave-one-out plot for MR analysis of CUD on PDs. (A) CUD on OCPD (B) CUD on APD (C) CUD on DPD (D) CUD on ASPD (E) CUD on EUPD (F) CUD on HPD (G) CUD on PPD (H) CUD on SPD (I) CUD on PD. PD, personality disorders; OCPD, anankastic (obsessive–compulsive) personality disorder; APD, anxious personality disorder; DPD, dependent personality disorder; ASPD, antisocial personality disorder; EUPD, emotionally unstable personality disorder; HPD, histrionic personality disorder; PPD, paranoid personality disorder; SPD, schizoid personality disorder; CUD, cannabis use disorder.


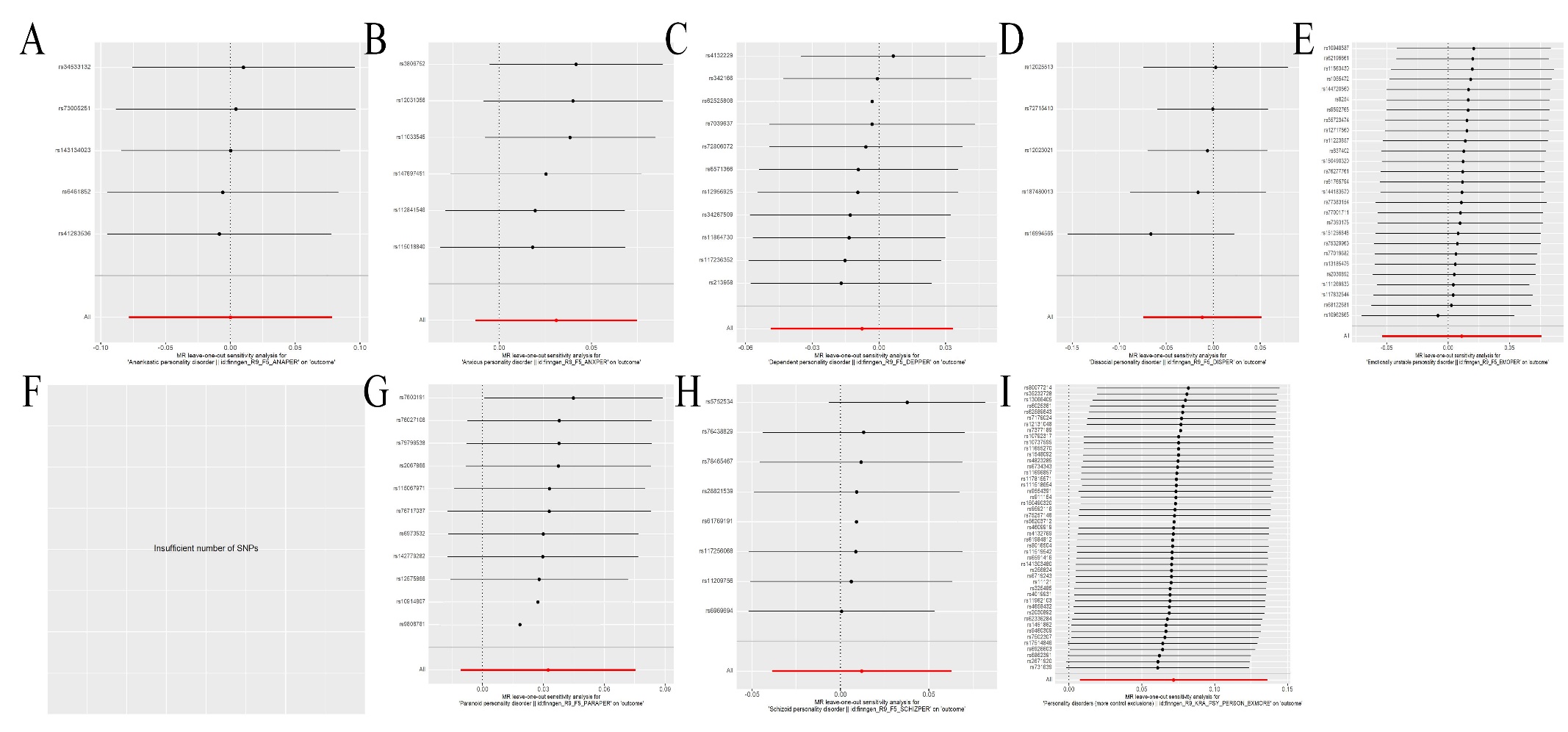


**Supplementary Fig. 3** Leave-one-out plot for MR analysis of PDs on CUD. (A) OCPD on CUD (B) APD on CUD (C) DPD on CUD (D) ASPD on CUD (E) EUPD on CUD (F) HPD on CUD (G) PPD on CUD (H) SPD on CUD (I) PD on CUD. PD, personality disorders; OCPD, anankastic (obsessive–compulsive) personality disorder; APD, anxious personality disorder; DPD, dependent personality disorder; ASPD, antisocial personality disorder; EUPD, emotionally unstable personality disorder; HPD, histrionic personality disorder; PPD, paranoid personality disorder; SPD, schizoid personality disorder; CUD, cannabis use disorder.


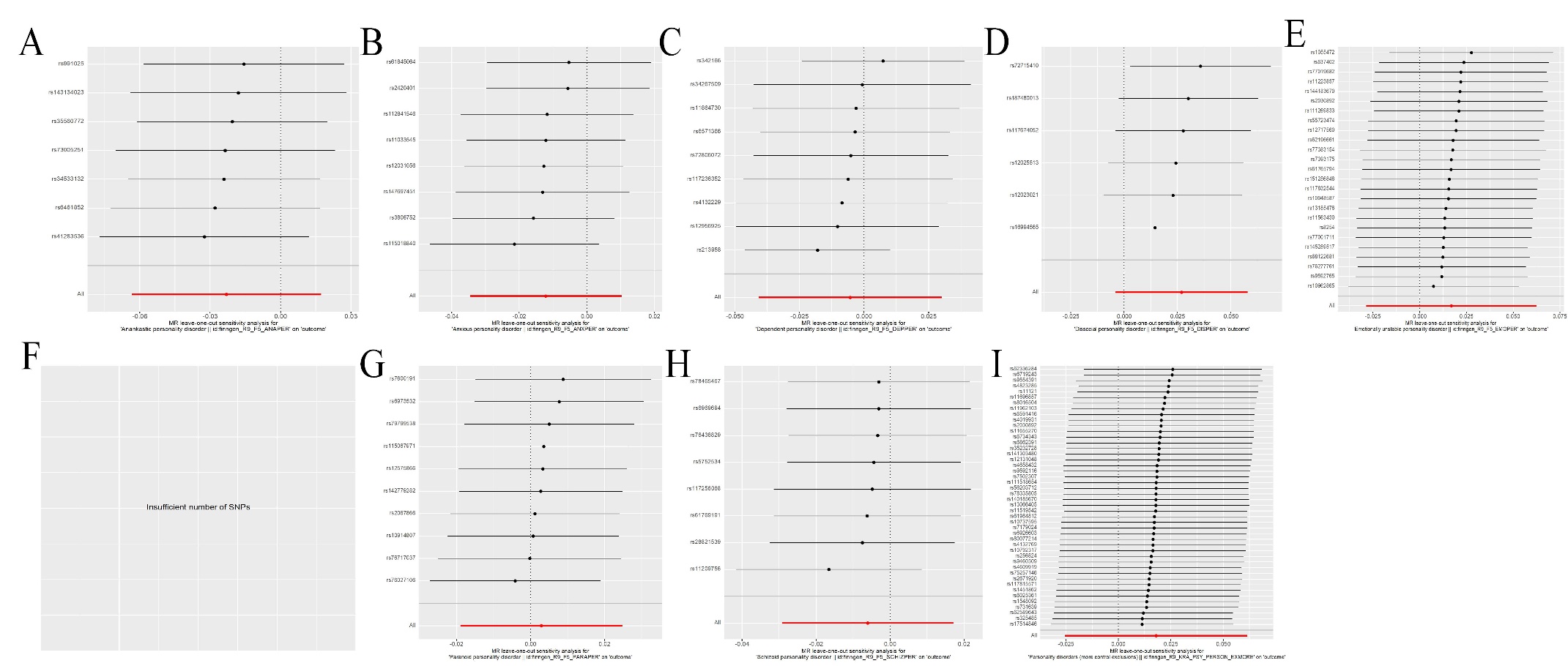


**Supplementary Fig. 4** Leave-one-out plot for MR analysis of PDs on LCU. (A) OCPD on LCU (B) APD on LCU (C) DPD on LCU (D) ASPD on LCU (E) EUPD on LCU (F) HPD on LCU (G) PPD on LCU (H) SPD on LCU (I) PD on LCU. PD, personality disorders; OCPD, anankastic (obsessive–compulsive) personality disorder; APD, anxious personality disorder; DPD, dependent personality disorder; ASPD, antisocial personality disorder; EUPD, emotionally unstable personality disorder; HPD, histrionic personality disorder; PPD, paranoid personality disorder; SPD, schizoid personality disorder; LCU, lifetime cannabis use.


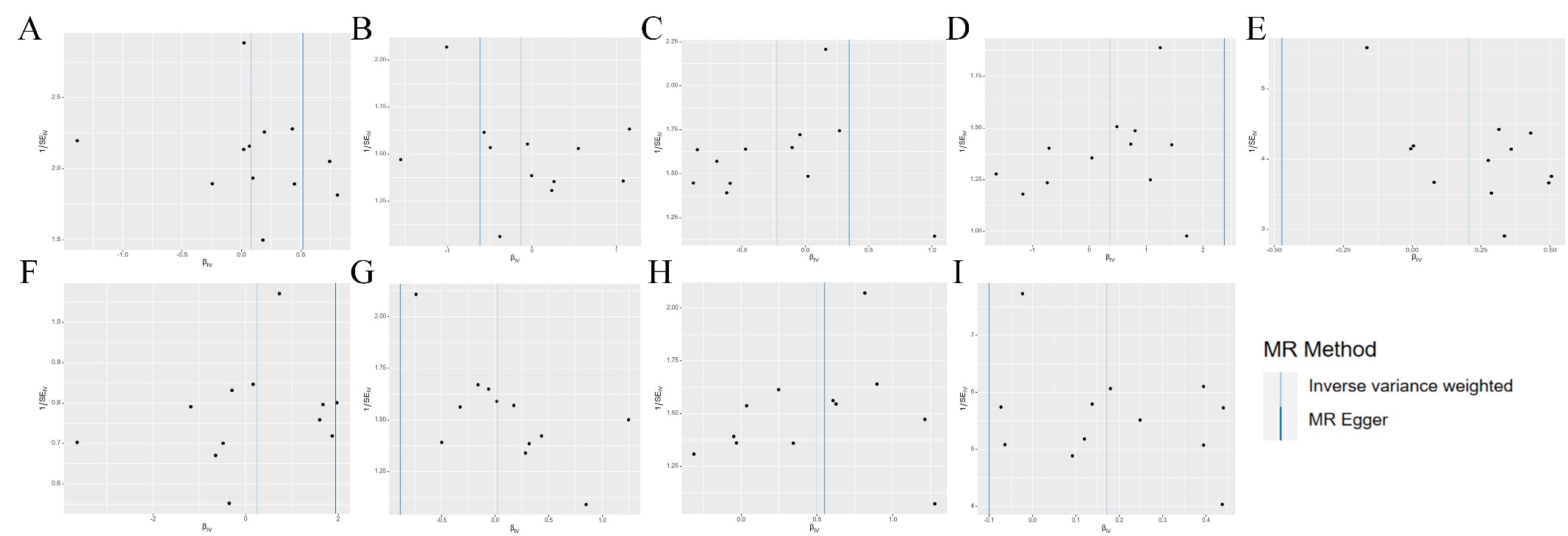


**Supplementary Fig. 5 Funnel plot of instrument precision against instrumental variable estimates for each genetic variant separately for Mendelian randomization analysis of** **CUD on PDs risk.** (A) CUD on OCPD (B) CUD on APD (C) CUD on DPD (D) CUD on ASPD (E) CUD on EUPD (F) CUD on HPD (G) CUD on PPD (H) CUD on SPD (I) CUD on PD. PD, personality disorders; OCPD, anankastic (obsessive–compulsive) personality disorder; APD, anxious personality disorder; DPD, dependent personality disorder; ASPD, antisocial personality disorder; EUPD, emotionally unstable personality disorder; HPD, histrionic personality disorder; PPD, paranoid personality disorder; SPD, schizoid personality disorder; CUD, cannabis use disorder.


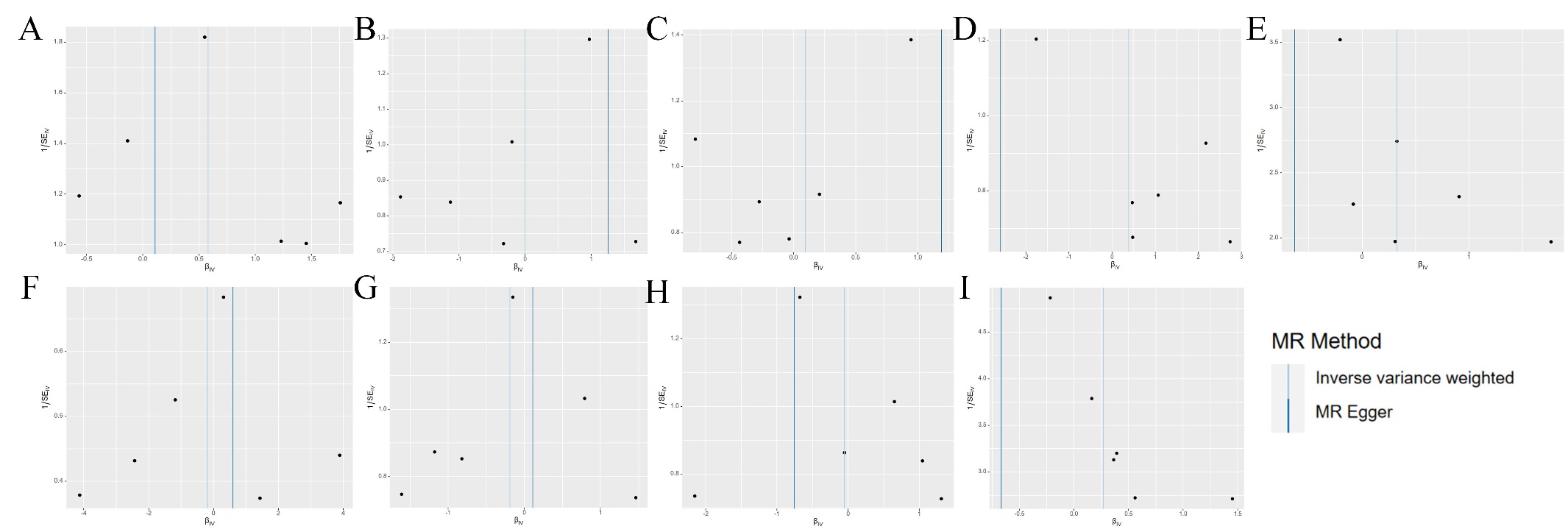


**Supplementary Fig. 6 Funnel plot of instrument precision against instrumental variable estimates for each genetic variant separately for Mendelian randomization analysis of LCU on PDs risk.** (A) LCU on OCPD (B) LCU on APD (C) LCU on DPD (D) LCU on ASPD (E) LCU on EUPD (F) LCU on HPD (G) LCU on PPD (H) LCU on SPD (I) LCU on PD. PD, personality disorders; OCPD, anankastic (obsessive–compulsive) personality disorder; APD, anxious personality disorder; DPD, dependent personality disorder; ASPD, antisocial personality disorder; EUPD, emotionally unstable personality disorder; HPD, histrionic personality disorder; PPD, paranoid personality disorder; SPD, schizoid personality disorder; LCU, lifetime cannabis use.


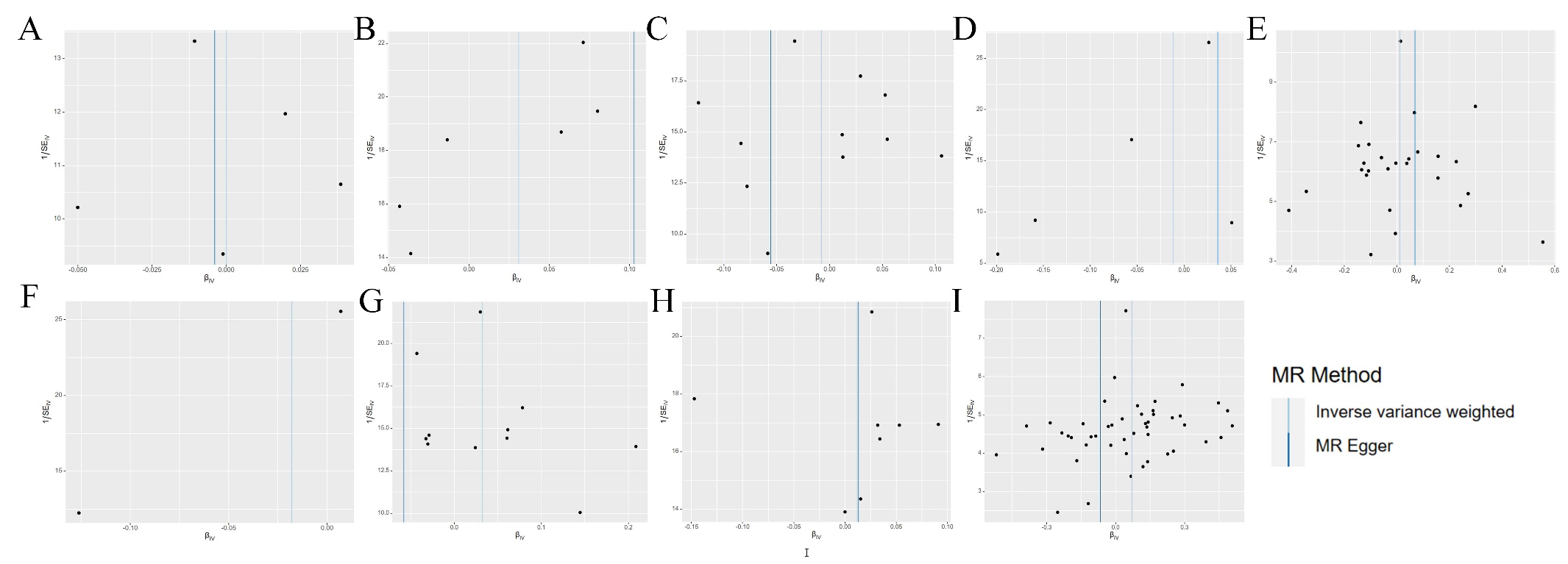


**Supplementary Fig. 7 Funnel plot of instrument precision against instrumental variable estimates for each genetic variant separately for Mendelian randomization analysis of PDs on CUD risk.** (A) OCPD on CUD (B) APD on CUD (C) DPD on CUD (D) ASPD on CUD (E) EUPD on CUD (F) HPD on CUD (G) PPD on CUD (H) SPD on CUD (I) PD on CUD. PD, personality disorders; OCPD, anankastic (obsessive–compulsive) personality disorder; APD, anxious personality disorder; DPD, dependent personality disorder; ASPD, antisocial personality disorder; EUPD, emotionally unstable personality disorder; HPD, histrionic personality disorder; PPD, paranoid personality disorder; SPD, schizoid personality disorder; CUD, cannabis use disorder.


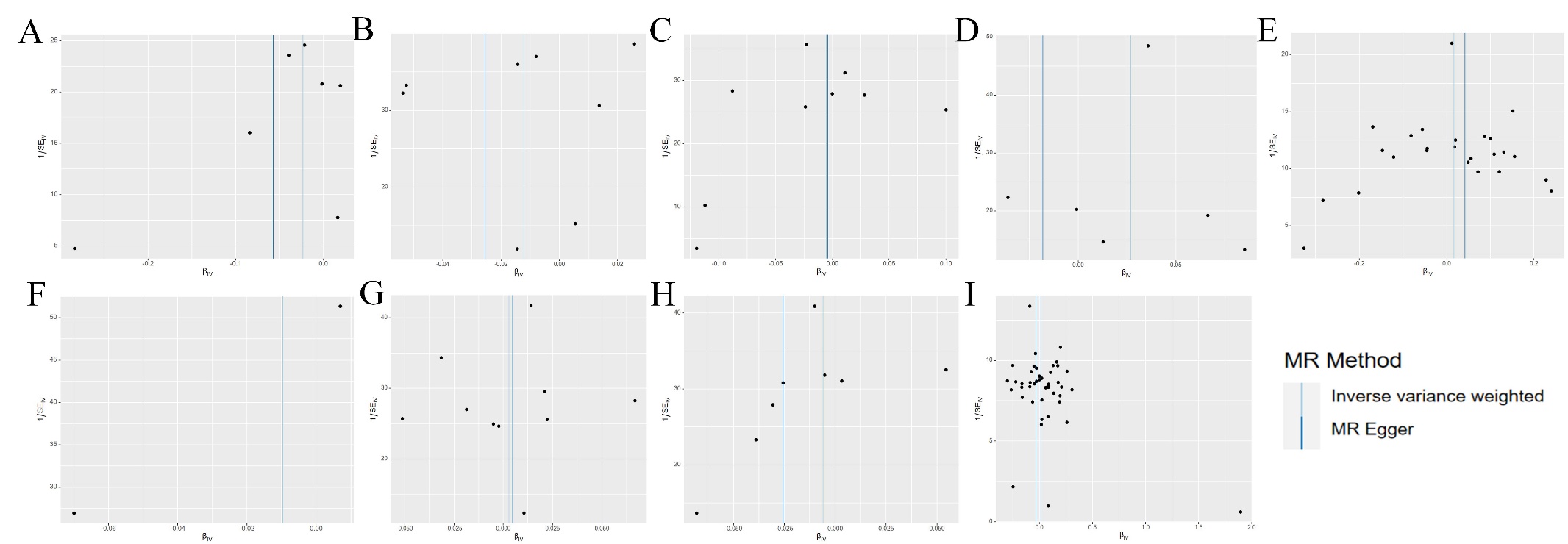


**Supplementary Fig. 8 Funnel plot of instrument precision against instrumental variable estimates for each genetic variant separately for Mendelian randomization analysis of PDs on LCU risk.** (A) OCPD on LCU (B) APD on LCU (C) DPD on LCU (D) ASPD on LCU (E) EUPD on LCU (F) HPD on LCU (G) PPD on LCU (H) SPD on LCU (I) PD on LCU. PD, personality disorders; OCPD, anankastic (obsessive–compulsive) personality disorder; APD, anxious personality disorder; DPD, dependent personality disorder; ASPD, antisocial personality disorder; EUPD, emotionally unstable personality disorder; HPD, histrionic personality disorder; PPD, paranoid personality disorder; SPD, schizoid personality disorder; LCU, lifetime cannabis use.


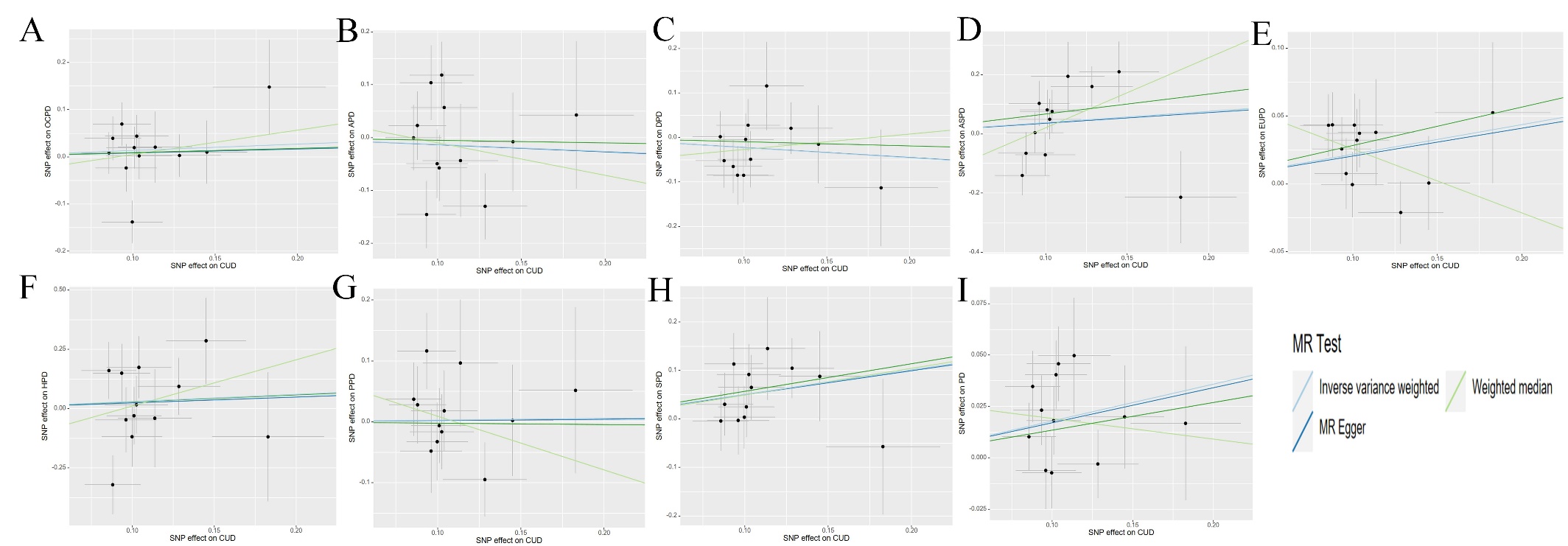


**Supplementary Fig. 9** Genetic associations with CUD (horizontal axis, standard deviation units) and with PDs (vertical axis, log odds ratios) at a genome- wide level of significance. (A) CUD on OCPD (B) CUD on APD (C) CUD on DPD (D) CUD on ASPD (E) CUD on EUPD (F) CUD on HPD (G) CUD on PPD (H) CUD on SPD (I) CUD on PD. PD, personality disorders; OCPD, anankastic (obsessive–compulsive) personality disorder; APD, anxious personality disorder; DPD, dependent personality disorder; ASPD, antisocial personality disorder; EUPD, emotionally unstable personality disorder; HPD, histrionic personality disorder; PPD, paranoid personality disorder; SPD, schizoid personality disorder; CUD, cannabis use disorder. Horizontal and vertical lines represent 95% confidence intervals for the genetic associations. The regression line through the origin represents the inverse-variance weighted Mendelian randomization estimate for the effect of effect of CUD on PDs.


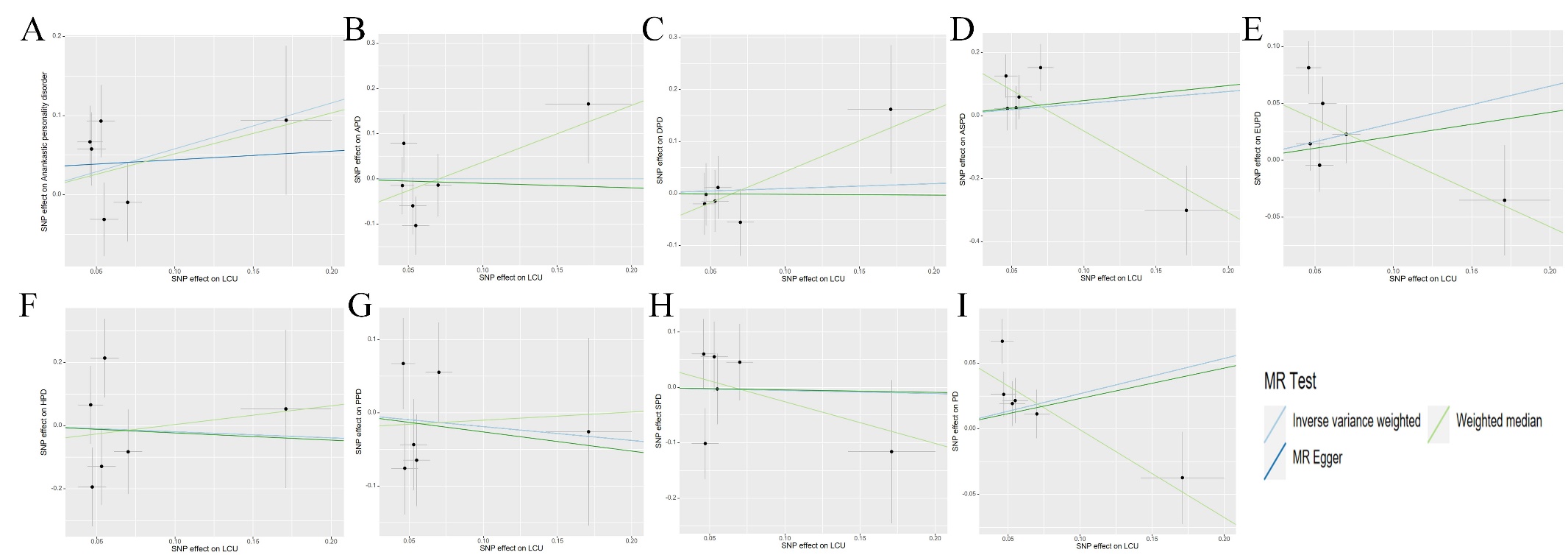


**Supplementary Fig. 10** Genetic associations with LCU (horizontal axis, standard deviation units) and with PDs (vertical axis, log odds ratios) at a genome- wide level of significance. (A) LCU on OCPD (B) LCU on APD (C) LCU on DPD (D) LCU on ASPD (E) LCU on EUPD (F) LCU on HPD (G) LCU on PPD (H) LCU on SPD (I) LCU on PD. PD, personality disorders; OCPD, anankastic (obsessive–compulsive) personality disorder; APD, anxious personality disorder; DPD, dependent personality disorder; ASPD, antisocial personality disorder; EUPD, emotionally unstable personality disorder; HPD, histrionic personality disorder; PPD, paranoid personality disorder; SPD, schizoid personality disorder; LCU, lifetime cannabis use. Horizontal and vertical lines represent 95% confidence intervals for the genetic associations. The regression line through the origin represents the inverse-variance weighted Mendelian randomization estimate for the effect of effect of LCU on PDs.


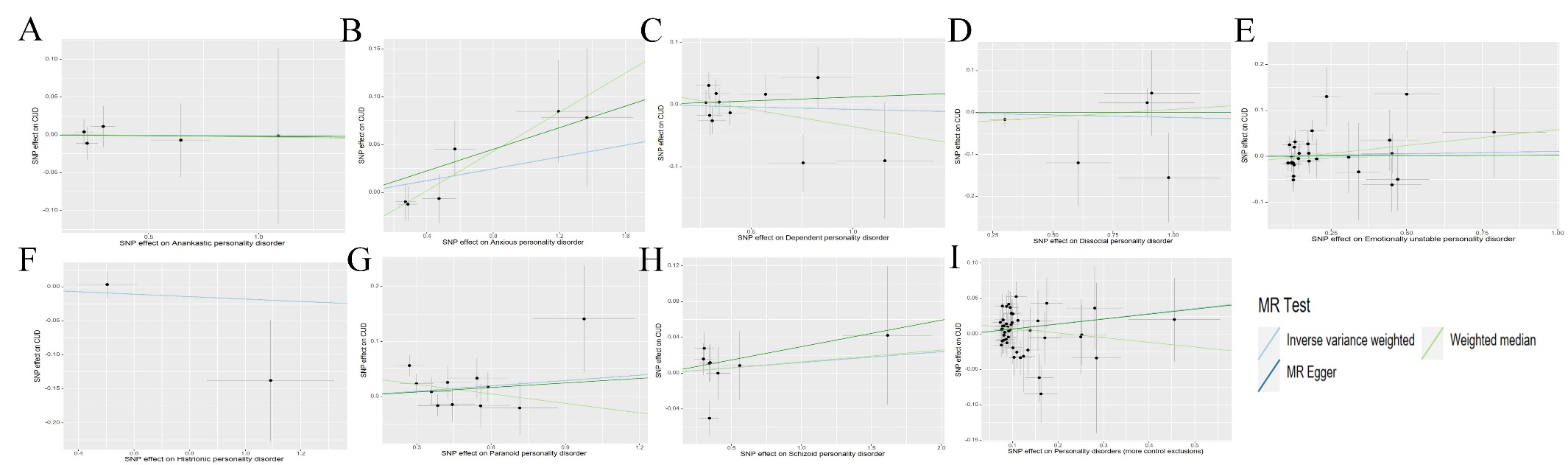


**Supplementary Fig. 11** Genetic associations with PDs (horizontal axis, standard deviation units) and with CUD (vertical axis, log odds ratios) at a genome- wide level of significance. (A) OCPD on CUD (B) APD on CUD (C) DPD on CUD (D) ASPD on CUD (E) EUPD on CUD (F) HPD on CUD (G) PPD on CUD (H) SPD on CUD (I) PD on CUD. PD, personality disorders; OCPD, anankastic (obsessive–compulsive) personality disorder; APD, anxious personality disorder; DPD, dependent personality disorder; ASPD, antisocial personality disorder; EUPD, emotionally unstable personality disorder; HPD, histrionic personality disorder; PPD, paranoid personality disorder; SPD, schizoid personality disorder; CUD, cannabis use disorder. Horizontal and vertical lines represent 95% confidence intervals for the genetic associations. The regression line through the origin represents the inverse-variance weighted Mendelian randomization estimate for the effect of effect of PDs on CUD.


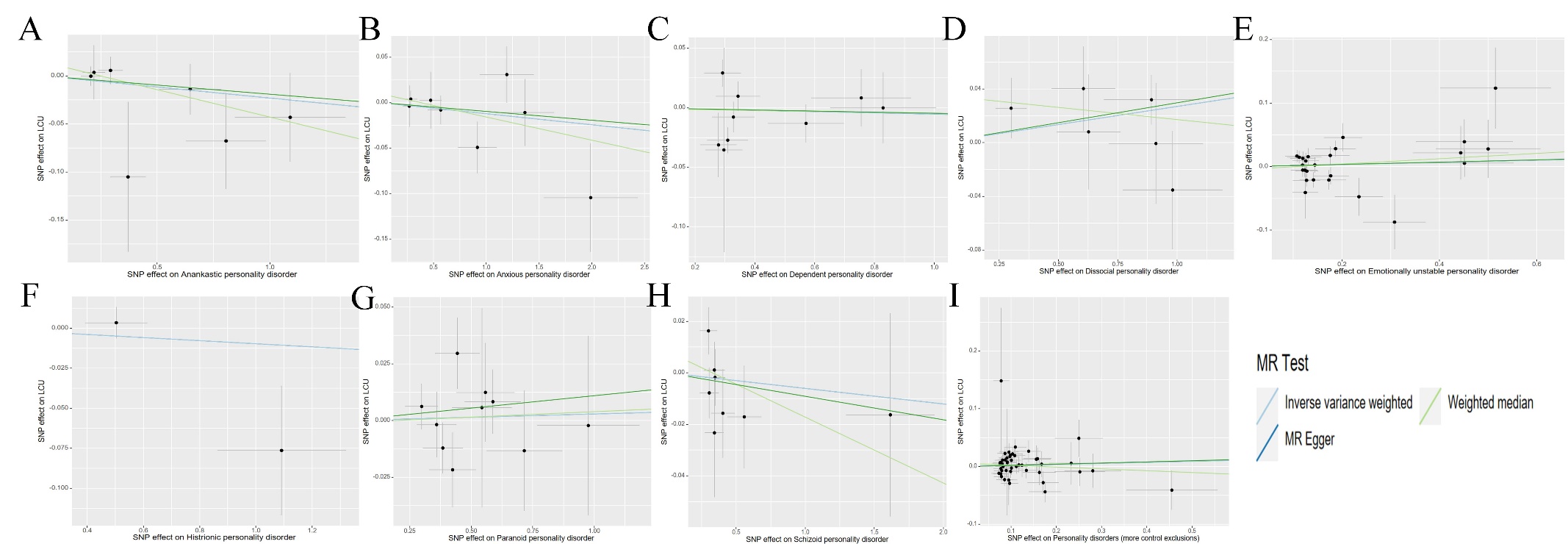


**Supplementary Fig. 12** Genetic associations with PDs (horizontal axis, standard deviation units) and with LCU (vertical axis, log odds ratios) at a genome- wide level of significance. (A) OCPD on LCU (B) APD on LCU (C) DPD on LCU (D) ASPD on LCU (E) EUPD on LCU (F) HPD on LCU (G) PPD on LCU (H) SPD on LCU (I) PD on LCU. PD, personality disorders; OCPD, anankastic (obsessive–compulsive) personality disorder; APD, anxious personality disorder; DPD, dependent personality disorder; ASPD, antisocial personality disorder; EUPD, emotionally unstable personality disorder; HPD, histrionic personality disorder; PPD, paranoid personality disorder; SPD, schizoid personality disorder; LCU, lifetime cannabis use. Horizontal and vertical lines represent 95% confidence intervals for the genetic associations. The regression line through the origin represents the inverse-variance weighted Mendelian randomization estimate for the effect of effect of PDs on LCU.


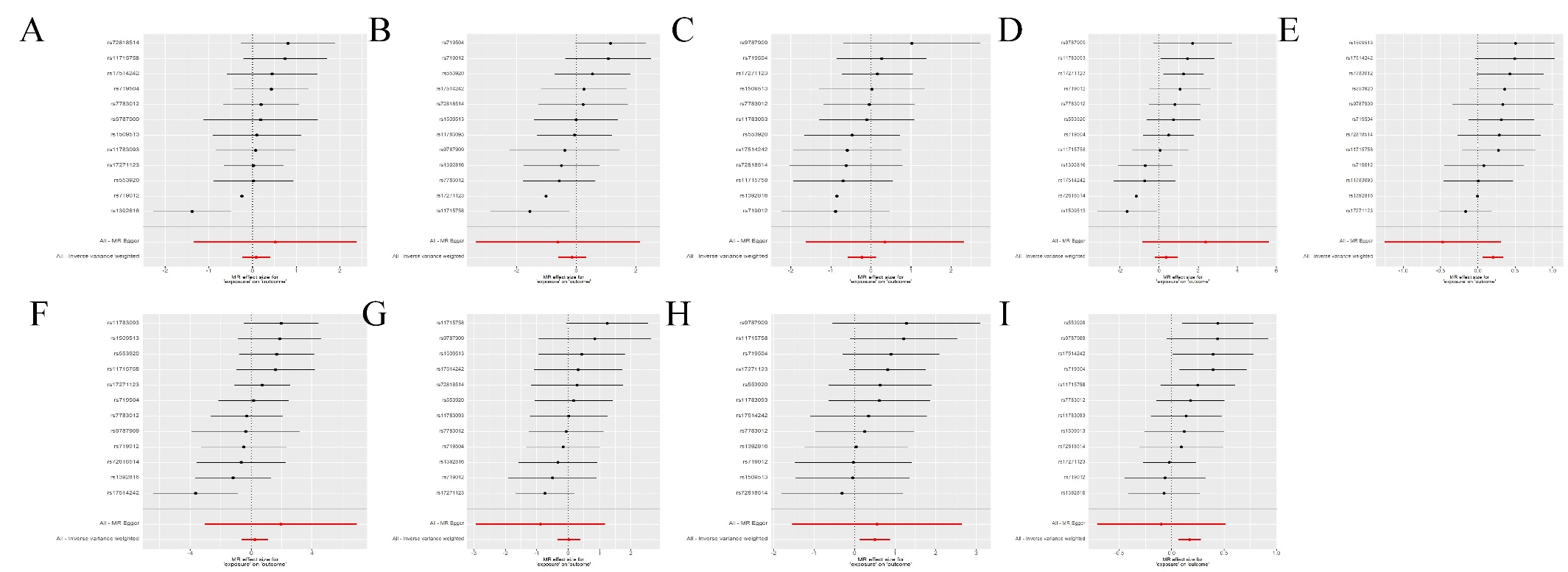


**Supplementary Fig. 13** Single-SNP analysis forest plots of the effect of CUD on PDs phenotypes. Point estimates represent the variant-specific ratio estimates for each SNP (in black), and the inverse-variance weighted (IVW) estimate (in red). Horizontal lines represent 95% confidence intervals around the variant-specific ratio estimates and the IVW estimate. (A) CUD on OCPD (B) CUD on APD (C) CUD on DPD (D) CUD on ASPD (E) CUD on EUPD (F) CUD on HPD (G) CUD on PPD (H) CUD on SPD (I) CUD on PD. PD, personality disorders; OCPD, anankastic (obsessive–compulsive) personality disorder; APD, anxious personality disorder; DPD, dependent personality disorder; ASPD, antisocial personality disorder; EUPD, emotionally unstable personality disorder; HPD, histrionic personality disorder; PPD, paranoid personality disorder; SPD, schizoid personality disorder; CUD, cannabis use disorder.


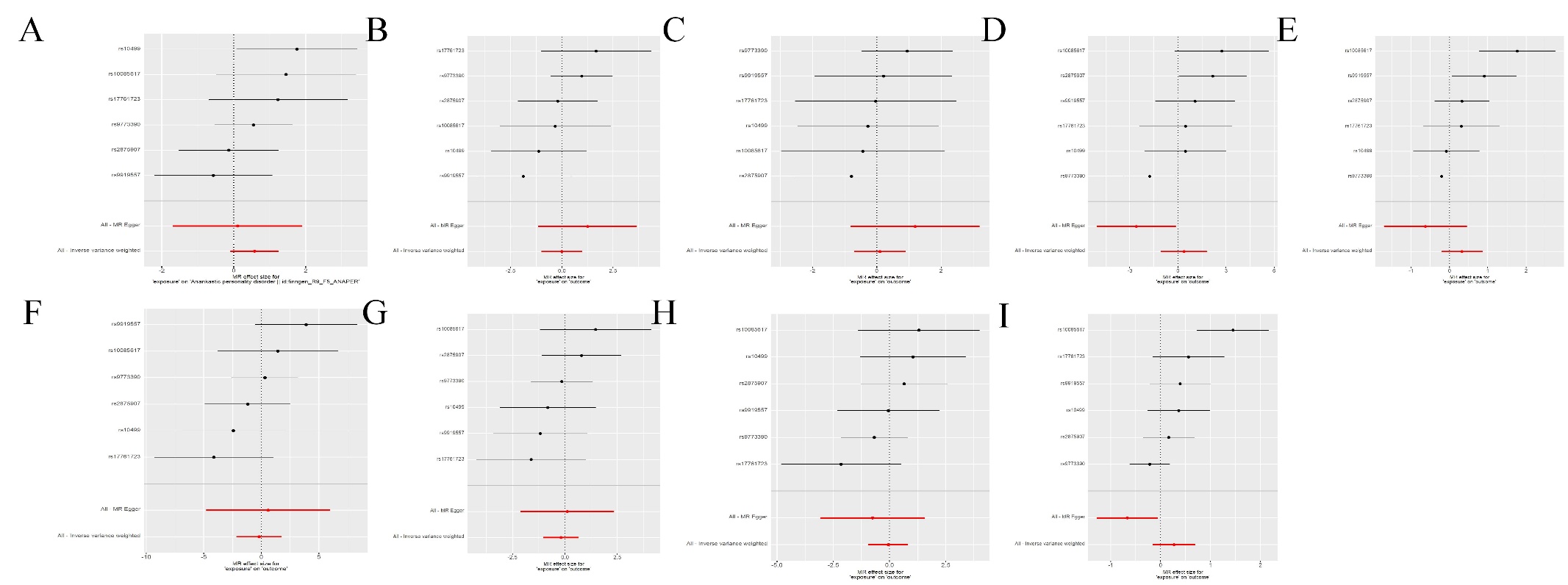


**Supplementary Fig. 14** Single-SNP analysis forest plots of the effect of LCU on PDs phenotypes. Point estimates represent the variant-specific ratio estimates for each SNP (in black), and the inverse-variance weighted (IVW) estimate (in red). Horizontal lines represent 95% confidence intervals around the variant-specific ratio estimates and the IVW estimate. (A) LCU on OCPD (B) LCU on APD (C) LCU on DPD (D) LCU on ASPD (E) LCU on EUPD (F) LCU on HPD (G) LCU on PPD (H) LCU on SPD (I) LCU on PD. PD, personality disorders; OCPD, anankastic (obsessive–compulsive) personality disorder; APD, anxious personality disorder; DPD, dependent personality disorder; ASPD, antisocial personality disorder; EUPD, emotionally unstable personality disorder; HPD, histrionic personality disorder; PPD, paranoid personality disorder; SPD, schizoid personality disorder; LCU, lifetime cannabis use.


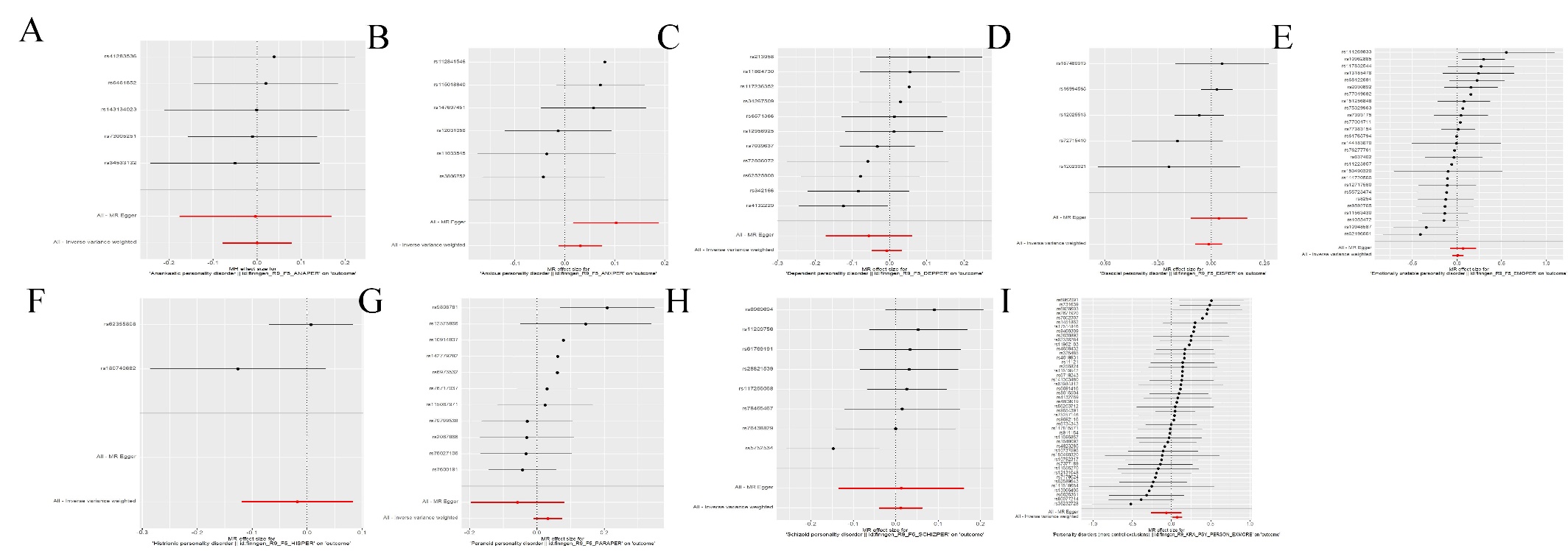


**Supplementary Fig. 15** Single-SNP analysis forest plots of the effect of PDs on CUD phenotypes. Point estimates represent the variant-specific ratio estimates for each SNP (in black), and the inverse-variance weighted (IVW) estimate (in red). Horizontal lines represent 95% confidence intervals around the variant-specific ratio estimates and the IVW estimate. (A) OCPD on CUD (B) APD on CUD (C) DPD on CUD (D) ASPD on CUD (E) EUPD on CUD (F) HPD on CUD (G) PPD on CUD (H) SPD on CUD (I) PD on CUD. PD, personality disorders; OCPD, anankastic (obsessive–compulsive) personality disorder; APD, anxious personality disorder; DPD, dependent personality disorder; ASPD, antisocial personality disorder; EUPD, emotionally unstable personality disorder; HPD, histrionic personality disorder; PPD, paranoid personality disorder; SPD, schizoid personality disorder; CUD, cannabis use disorder.


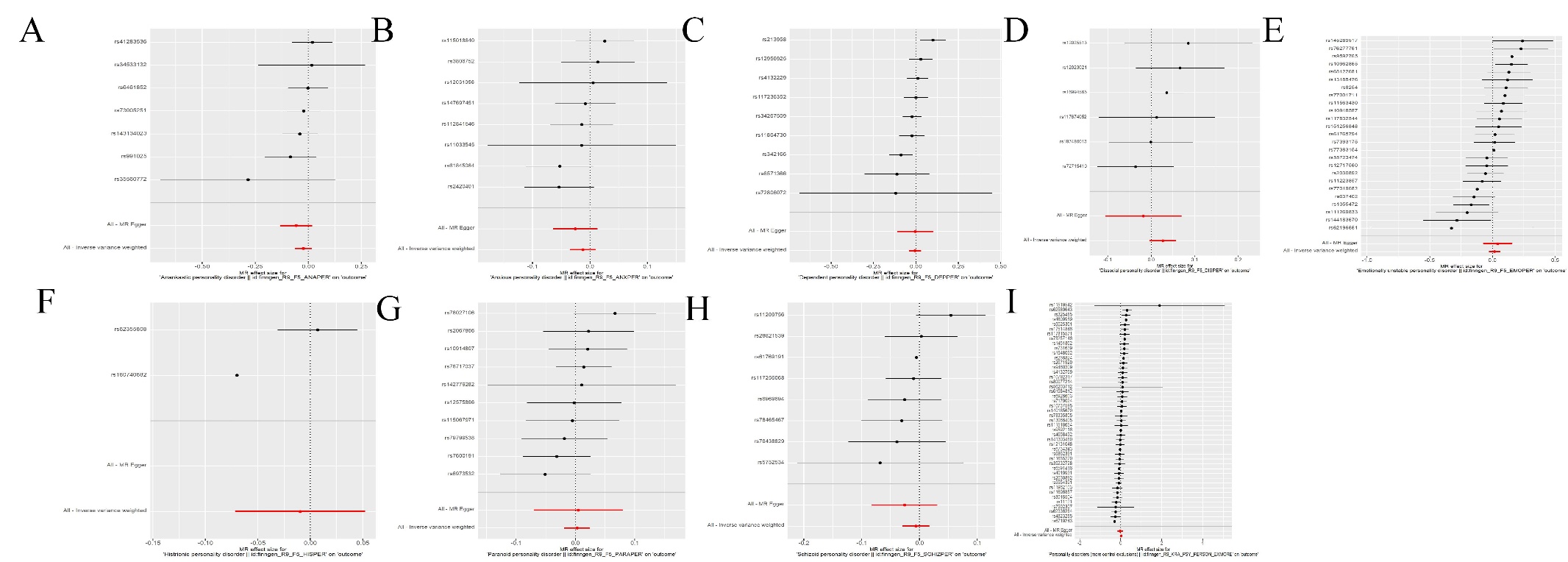


**Supplementary Fig. 16** Single-SNP analysis forest plots of the effect of PDs on CUD phenotypes. Point estimates represent the variant-specific ratio estimates for each SNP (in black), and the inverse-variance weighted (IVW) estimate (in red). Horizontal lines represent 95% confidence intervals around the variant-specific ratio estimates and the IVW estimate. (A) OCPD on LCU (B) APD on LCU (C) DPD on LCU (D) ASPD on LCU (E) EUPD on LCU (F) HPD on LCU (G) PPD on LCU (H) SPD on LCU (I) PD on LCU. PD, personality disorders; OCPD, anankastic (obsessive–compulsive) personality disorder; APD, anxious personality disorder; DPD, dependent personality disorder; ASPD, antisocial personality disorder; EUPD, emotionally unstable personality disorder; HPD, histrionic personality disorder; PPD, paranoid personality disorder; SPD, schizoid personality disorder; LCU, lifetime cannabis use.


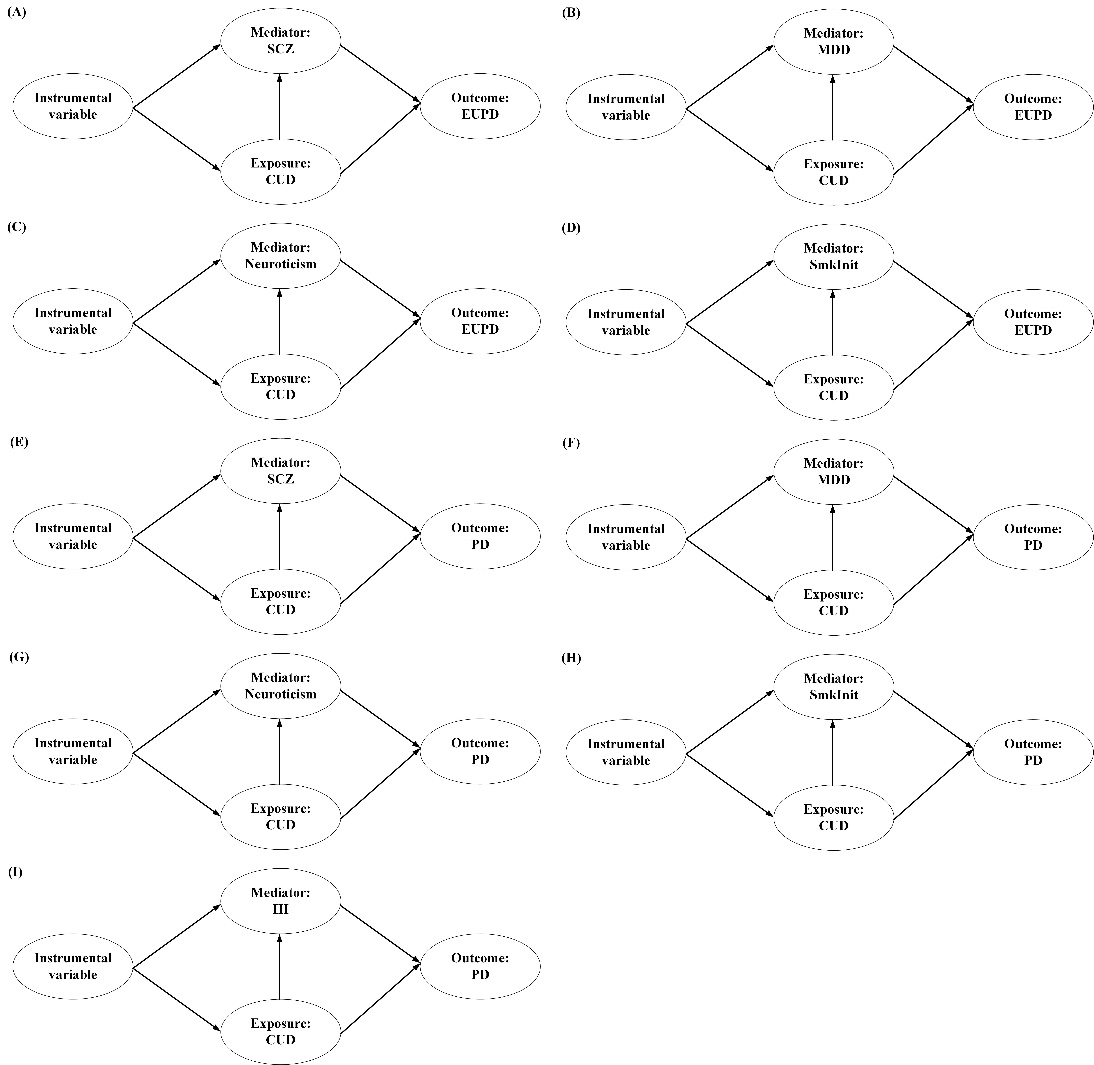


**Supplementary Fig. 17 A directed acyclic graph (DAG) illustrating the relationships between cannabis use, mediators, and the outcomes.** PD, personality disorders; EUPD, emotionally unstable personality disorder; CUD, cannabis use disorder; SCZ, schizophrenia; SmkInit, smoking initiation; HI, household income; MDD, major depressive disorder.
